# Heavy rainfalls in Poland and their hyetographs

**DOI:** 10.1007/s13280-024-02069-6

**Published:** 2024-09-16

**Authors:** Karol Mikołajewski, Alfred Stach, Marek Ruman, Klaudia Kosek, Zbigniew W. Kundzewicz, Paweł Licznar

**Affiliations:** 1 RETENCJAPL Sp. Z o.o., Marynarki Polskiej 163, 80-868 Gdańsk, Poland; 2 Faculty of Natural Sciences, University of Silesia in Katowice, Będzińska 60, 41-200 Sosnowiec, Poland; 3 Department of Geoinformation, Faculty of Geographical and Geological Sciences, Adam Mickiewicz University in Poznań, B. Krygowskiego 10, 61-680 Poznań, Poland; 4 Marine Chemistry and Biochemistry Department, Institute of Oceanology, Polish Academy of Sciences, Powstańców Warszawy 55, 81-712 Sopot, Poland; 5 Faculty of Environmental Engineering and Mechanical Engineering, Poznan University of Life Sciences, Piątkowska 94b, 60-649 Poznań, Poland; 6 Faculty of Building Services, Hydro and Environmental Engineering, Warsaw University of Technology, Nowowiejska 20, 00-653 Warsaw, Poland

**Keywords:** Classification quality assessment indices, Cluster analysis, Heavy rainfalls, Model hyetographs, Poland, Precipitation modelling

## Abstract

In the light of observed variability in precipitation patterns, there is a growing need for comprehensive data mining of regularly updated rainfall recording databases. Therefore, an analysis of heavy rainfall and hyetographs was conducted using a 30-year high-resolution dataset from 100 rain gauges across Poland, covering 31 646 rainfall events. Distributions of rainfall depths, durations, and intensities were explored, and maxima were compared to global records. Spatial analysis revealed significant variations in the frequency, depths, and durations of extreme rainfall across different regions. Cluster analysis determined model hyetographs for each station. The likelihood of regions belonging to clusters with three to five model hyetographs was assessed using Indicator Kriging. Findings underscore the importance of using local, characteristics rainfalls in hydrodynamic modelling of drainage systems and future rainfall scenarios. These results provide a foundational step towards understanding and monitoring the impacts of climate change on rainfall characteristics, especially extremes, in future decades.

**Supplementary Information:**

The online version contains supplementary material available at 10.1007/s13280-024-02069-6.

## Introduction

In recent years, the world has witnessed a significant shift in the occurrence of extreme rainfall events, driven by ongoing and intensifying climate change. These changes in precipitation patterns have profound implications for various regions, including Poland. Characterising the prevailing rainfalls in Poland, it can be stated that they are primarily occurring during the movement of frontal zones from the Atlantic Ocean. Their efficiency is higher when storms form in the zone of cold fronts. Relatively short-lived but intense rainfall is associated with convection within a homogeneous, moist air mass. Their spatial distribution is most often random and has no clear connection with the terrain. However, maximum rainfall totals have a different origin. They occur in the Carpathians and Sudetes Mountains during a northern cyclonic situation, when moist air masses accumulate on the windward slopes of the mountains. In Poland, rains of high efficiency and intensity most often occur in June and July, accounting for over 60% of cases. The natural water cycle in Poland, like in many other parts of the globe, has experienced disruption due to rapid changes in land use and land cover, primarily stemming from urbanisation and the extensive transformation of agricultural areas into built-up zones (Tanaś and Trojanek 2014; Castanho et al. 2019; Gargula et al. 2020; SOER 2020). One notable consequence of this transformation is the increasing surface sealing, which hinders rainfall from infiltrating into the ground. Consequently, the retention capacity of built-up areas diminishes, and the runoff coefficient rises. As rainwater cannot percolate into the soil, it instead flows over paved surfaces, potentially culminating in violent surface runoff. This alteration in the natural water cycle has far-reaching implications for urban areas and their vulnerability to flooding.

However, the challenges posed by shifting land use and urbanisation are compounded by the broader context of climate change. Climate change is not solely synonymous with rising temperatures; it also entails shifts in the spatial and temporal patterns of extreme precipitation events. These changes encompass an increase in both the intensity and frequency of heavy rainfalls on a global scale (Seneviratne et al. 2021), a trend similarly observed in numerous European regions and countries (Madsen et al. 2014). This phenomenon aligns with the Clausius–Clapeyron equation, which describes the relationship between saturated vapour pressure and temperature, elucidating that a warmer atmosphere can accommodate more water vapour, potentially leading to more intense precipitation (Kundzewicz and Pińskwar 2022).

Furthermore, formal detection and attribution analyses (Pall et al. 2011) have unveiled a noteworthy contribution of anthropogenic greenhouse gas emissions to the intensification of heavy precipitation. Consequently, the consequences of increased intense precipitation are evident, particularly in the form of pluvial floods—flash and urban floods caused by heavy rainfalls—which have surged in both frequency and magnitude. According to projections for the future, the load is likely to aggravate, not only due to increasing urbanisation, but also due to climate change. Even if the nature of changes of extreme precipitation in the warming climate of Poland is complex (Pińskwar 2022), the occurrence of long intervals with low precipitation interspersed by episodes of increasingly heavy rainfall can be expected. The system's resistance should therefore be increased to match the increasing load. Hence, it is becoming necessary to upgrade municipal rainwater drainage systems so that they can accommodate greater loads (Kundzewicz and Licznar 2021). Rainwater drainage systems are a key element of the critical municipal infrastructure. The tasks they are facing today are not only limited to the interception and discharge of rainwater to nearby receivers, i.e. surface water bodies. There is an increasing need to store water in drainage systems. Storage reservoirs built on rainwater drainage systems are intended not only to simply slow down the runoff of rainwater, but also to restore and compensate for the water retention lost due to sealing of natural surfaces. Water retention should be of a long-term nature, and the rainwater captured in such storage reservoirs should be treated as a resource for use by city dwellers. Measures increasing the retention of drainage systems aim at reducing not only the maximum runoff caused by rainwater, but also the total runoff volume. Active management of rainwater is also becoming increasingly important. According to the smart cities concept, drainage systems and their retention resources should be managed intelligently. The growing role of drainage systems and the need to restore rainwater retention pose new challenges related to the modernisation of the toolbox of engineering design and modelling. New tools and data sources have already emerged. The design of drainage systems is increasingly frequently carried out in specialised computer applications using digital maps, GIS resources, and digital terrain models.

Despite the vivid consequences of climate change on precipitation dynamics, the assumption of stationarity, i.e. temporal invariance of the frequency of annual maximum daily precipitation, is commonly used in practice for designing infrastructure: storm sewers, roads, railways, bridges, and culverts (Kundzewicz and Licznar 2021). The concept of design rainfall is of crucial importance in natural hazard risk reduction, water management, and climate change adaptation. The engineering design standards serve as the basis of both designing the infrastructure and perception of tolerable risk. Only in selected countries is the awareness of climate change high enough to systematically update technical standards based on the latest observational data on precipitation or even make projections based on the results of climate models (Kundzewicz and Licznar 2021). The determination of design rainfall requires information on the greatest observed point rainfalls, probable maximum precipitation, as well as intensity–duration–frequency–area and depth–duration–frequency–area relations (Markiewicz 2021). The advent of urban runoff models brought new demands on hydroclimatology, namely the requirement of information on the time-distribution characteristics of rainfall during heavy storms. This method is recommended for computing runoff, particularly through rainfall–runoff models used in designing and operating runoff control structures, as well as for assessing individual storm events after they occur. It is important to note that variations in time-distribution models can significantly impact the results of runoff modelling for urban basins.

Bearing in mind the increasing frequency and severity of pluvial floods, flash floods, and urban floods, the understanding of the structure of the spatial–temporal fields of extreme precipitation in Poland needs improvement (Stach 2009). This includes the regional variability of characteristics of extreme precipitation, as well as the temporal change (that can be detected as a trend) and variability (deviations from the long-term trend, if such is detected). Moreover, the distribution of rainfall depth over the storm's duration needs to be determined.

The calculation of non-stationary surface storm runoff and its further transformation in the urban system requires scenarios of temporal distribution of a rainfall episode (Mikołajewski et al. 2022). Hydrological analyses require consideration of the temporal variability of point rainfall intensity, represented in the form of hyetographs. Adopting hyetographs in design aims at reflecting the temporal distribution of rainfall in recent past, and implicitly also potential rainfalls that may occur in the future, understood as the assumed duration of operation of the designed technical solutions. This is not a trivial task, however, not only because of the stochastic (random) nature of rainfall, but also of its multifractal nature (Deidda 2000; Gires et al. 2012). Until recently, the availability of accurate rainfall records at a high temporal resolution has typically been very limited. Hence, the historical methodology of model hyetograph development used to be based on generalisation and simplified assumptions without adequate validation. Interest in model hyetographs increased with first attempts of transition from stationary methods of calculation of urban stormwater systems. The model of stormwater runoff transformation in the drainage system can be combined with the hydrological surface runoff model, describing stormwater inflow to network nodes such as manholes and inlets from the catchment area and the stormwater system. The interaction of the stormwater system with the rainfall receiver could only be reflected by means of a suitable threshold condition on the network outlet.

Despite the currently growing access to precipitation time series records at a high temporal resolution, in hydrology, and particularly urban hydrology, engineers still model and design drainage systems using scenarios of temporal distributions of rainfall predefined by means of model hyetographs (Mikołajewski et al. 2022). Knowledge on how much rain can fall in a particular location in the country is in increasing demand. Regionalisation can be based on pooling similar or neighbouring stations together. The use of geostatistical tools allows for the estimation of the spatial field of precipitation depth with specific exceedance probabilities in locations for which there are no measurements (which allows for the extension of information and coverage of the entire country), but also for the estimation of the confidence intervals of these quantities. Because climate, land use, and land cover have changed in Poland in recent decades, and are projected to change in the future, reliable knowledge about properties of time-distribution of precipitation is needed for various spatial locations.

The occurring and forecasted climate changes and the resulting changes in recorded precipitation in Central Europe are manifested not so much in changes in average annual precipitation totals as in unfavourable prolongation of drought periods and occurrence of more seldom appearing, but more intensive rainfall. Proper monitoring of such transformations requires reference knowledge on heavy rainfalls from the period of recent decades. Considering the above, research was undertaken involving complex analyses of heavy rainfalls in Poland in terms of their basic characteristics and spatial distribution in the country, as well as regarding only temporal distributions, i.e. hyetograph models of recorded heavy rainfalls (Mikołajewski et al. 2022).

This study represents a pioneering effort, utilising a large and verified digital dataset of high-resolution precipitation data, and applying modern techniques such as geostatistical simulations and data mining. It aims to determine reference statistics for heavy rainfalls in Poland from 1986 to 2015 and develop methodologies for analysing extensive precipitation series from rain gauge networks. Pragmatically, it seeks to quantify the amount and duration of maximum heavy rainfalls and establish typical hyetographs, crucial for designing and modelling drainage systems in Poland.

By employing advanced geostatistical and data mining techniques, the study offers a robust methodological framework adaptable to regions experiencing similar precipitation shifts. Its comprehensive analysis of the temporal distribution of heavy rainfalls enhances understanding of hydrological processes, vital for creating resilient infrastructure and sustainable urban planning practices globally. This research underscores the need for updated engineering standards and proactive adaptation strategies, making it have a significant meaning to a readership widely interested in environmental sciences, urban planning, and climate change mitigation.

## Materials and methods

### Digital base of precipitation data

The study employed resources of the national precipitation base of the Polish Atlas of Rainfall Intensities (PANDa) Project. The base was developed in the period 2016–2017, and covered data from 30 years of precipitation records from 100 rain gauges in Poland (hence a total of 3000 station-years of observations), including synoptic stations (I and II order), climatic stations (III and IV order), and precipitation stations (V order). The list of all stations included in the digital base of the PANDa project with their geographic coordinates and heights above sea level is presented in Table S1 (Supplementary information). The preparation of the PANDa precipitation base employed analogue records (pluviograph strip charts) subject to digitisation with the application of a methodology proposed by Licznar et al. (2011), and digital records from electronic rain gauges. Analogue data in the form of pluviograph strip charts primarily covered observations of the warm hydrological half-year (from May to October), and digital data from electronic rain gauges already usually covered complete year-long records. Precipitation data recorded in the base adopt the form of time series with a standard temporal resolution of 1 min. All precipitation series in the base were verified in terms of accuracy, referring them to alternative precipitation records and analysing their structure with the application of methods of multifractal research. Detailed information regarding the PANDa digital precipitation base and its verification can be found in papers by Burszta-Adamiak et al. (2019) and Wilk et al. (2020).

The database includes values recorded between 1986 and 2015. To fill in gaps in the observation data, about 2% of the resources were supplemented with records from earlier years (dating back to 1980) and from the later year 2016. Approximately 40% of the data in the resulting PANDa database consists of year-round observations from electronic rain gauges. The remaining 60% is analogue data, which required digitising pluviographs from periods of the warm half-year.

### Designation of sets of heavy rainfalls

**Fig. 1:**
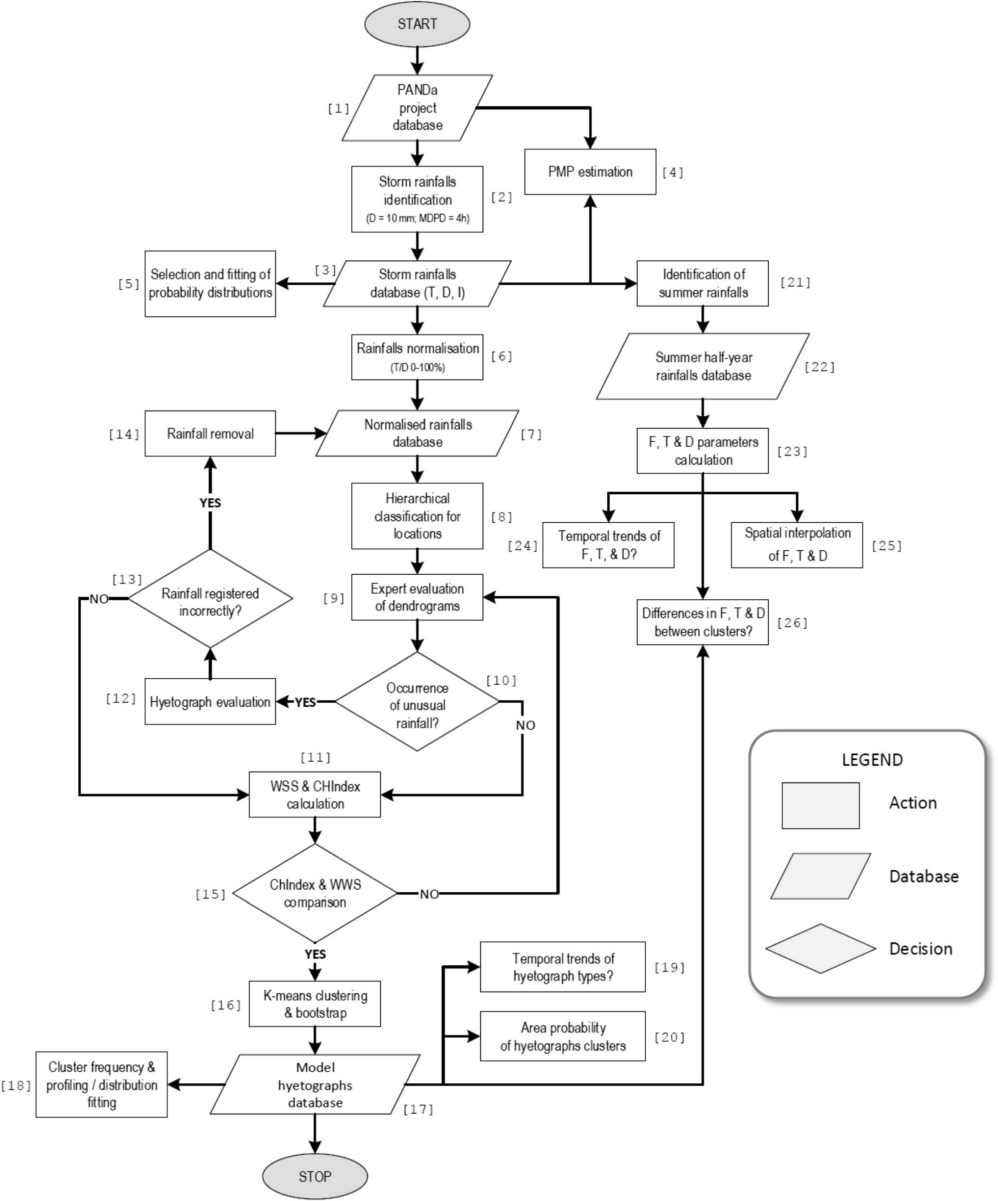
Flowchart of the entire process of creating standard hyetographs and additionally conducted data analysis. The numbers in square brackets next to the individual components of the diagram serve as references in the descriptions of the stages of the methodology in the text

Based on the cluster analysis methodology, the observed heavy rainfall sets were divided into rainfall clusters with similar temporal distributions, allowing for the final identification of local model hyetograph clusters. The cluster number was optimised in a rational, theoretically-justified way. The study deals with rainfall only (no snow), although in Poland snow may incidentally fall in late spring and early autumn, i.e. during the warm half-year. The methodology of the entire process, briefly described in this chapter, is presented in the diagram below as a flowchart—Fig. 1. References to individual blocks in this flowchart are included in the text of this and next subsection.

Heavy rainfalls were designated from precipitation time series of the PANDa database (Block [1] in Fig. 1) by criteria proposed by Wilk et al. (2020) for the identification of precipitation used for modelling municipal drainage systems. The criteria are a standard applied in Germany and Poland, and have been used, e.g. in papers written by Licznar et al. (2011, 2017) and Mikołajewski et al. (2022) (Block [2] in Fig. 1). A value of 10 mm was adopted as the threshold of total amount of heavy rainfall, and 4 h as the minimum time interval between individual rainfalls. These criteria were derived from the Commentary on the DWA-A-A118 guideline (Schmitt 2000). The foundation for this criterion was the results of research conducted by Wenzel and Voorhees (1981), which, in the context of urban watershed analysis, allowed for the estimation of the minimum break to distinguish independent cloudbursts over a period of 4–5 h. The MDPD (minimum dry–period duration) criterion according to the commentary on the DWA—A118 guidelines is frequently used in Poland (Licznar and Szelag 2014; Mikołajewski et al. 2022). The comparative rationale for adopting such an assumption is the similarity of climatic conditions in central Europe, the differentiation of rainfalls and periods with no rainfall adopted the minimum value of rainfall depth equal to 0.1 mm over 5 min as a threshold, so that the interval, due to the rainfall duration and depth, is considered as a part of the rainfall event.

Heavy rainfalls were designated automatically in the RainBrain Internet Base^1^ with the application of a specially prepared function. Table S1 (Supplementary information) which in addition to location data of measuring stations included in the PANDa database, also contains information on the number of heavy rainfalls distinguished on them, based on the criteria given above. The result of the designation of heavy rainfalls separately from rain gauges of the PANDa project was sets of heavy rainfalls. In sets of heavy rainfalls from particular stations, not only their total number and number in periods of the summer half-year was determined, but also total depths and durations (Block [3] in Fig. 1). The aforementioned parameters also provided the basis for the calculation of mean intensities of particular heavy rainfalls (expressed in mm min^−1^).

The selection and fitting of functions deQ3scribing the continuous distributions of histograms for duration, depth, and intensity at individual locations were done using EasyFit Pro v 5.5 software (Block [5] in Fig. 1). Over 30 theoretical continuous distributions were tested. The goodness of fit was assessed using Kolmogorov–Smirnov, Anderson–Darling, and Chi-squared tests. From the available distributions, gamma, exponential, and general extreme value were chosen. Those functions are frequently used in analyses of precipitation regimes. In each case, the empirical and theoretical distributions were found to be consistent at the alpha level < 0.01. Additionally, the fitting parameters of these distributions were determined for the entire dataset of all rainfall events. Detailed information about the used functions and their parameters is described below:

Distributions of heavy rainfall depth were modelled by means of the 2-parameter exponential distribution. The function of probability density of the distribution is described by the following equation:

1 $$f\left( x \right) = \lambda \cdot e^{{ - \lambda \left( {x - \gamma } \right)}} ,$$

where *λ*—inverse scale parameter (scale parameter is 1/*λ*). The parameter meets the condition *λ* > 0, *γ*—location parameter (for each rain gauge *γ* = 10, as resulting from the adopted criterion of designation of heavy rainfalls as precipitation with a depth higher or equal to 10 mm).

Distributions of values of total durations of heavy rainfalls were modelled with the application of the gamma distribution, given by the following probability density function:

2 $$f\left( x \right) = \frac{1}{{b^{a} \Gamma \left( a \right)}} \cdot x^{a - 1} \cdot e^{ - x/b} ,$$

where *a*—shape parameter (a > 0), Γ—gamma function, *b*—scale parameter (*b* > 0).

Distributions of values of average intensity of heavy rainfalls were modelled with the application of the generalised extreme value distribution (GEV). The function of probability density of the distribution for nonzero values of the shape parameter *k* (*k* ≠ 0) is defined as below:

3 $$f\left( x \right) = \left( {\frac{1}{\sigma }} \right) \cdot \exp \left( { - \left( {1 + k\frac{x - \mu }{\sigma }} \right)^{ - 1/k} } \right) \cdot \left( {1 + k\frac{x - \mu }{\sigma }} \right)^{{ - 1 - \frac{1}{k}}} ,$$

4 $${\text{for}}\;1 + k\frac{x - \mu }{\sigma } > 0,$$

and in the case of the shape parameter *k* = 0 with the following equation:

5 $$f\left( x \right) = \left( {\frac{1}{\sigma }} \right) \cdot \exp \left( { - \exp \left( { - \frac{x - \mu }{\sigma }} \right) - \frac{x - \mu }{\sigma }} \right),$$

where *k*—shape parameter, *σ*—scale parameter, *μ*—location parameter.

The parameters in the distribution Eqs. (1), (2), and (3) have been designated using common notations found in standard literature. Although different symbols are used to denote the scale, shape, and location parameters across different distributions, they refer to the respective properties of these distributions as described. For examples and further details, refer to Ross (2014), Wasserman (2004), or Rice (2006).

### Analysis of heavy rainfall hyetographs

Due to differing durations and depths of the designated heavy rainfalls, further analysis of the sets, i.e. mutual comparison and identification of typical (measurable, model) time distributions of heavy rainfalls, their cumulative hyetographs were converted into so-called dimensionless (normalised) cumulative hyetographs (Block [6] in Fig. 1). The conversion was performed in accordance with the methodology described in the publication by Licznar et al. (2017) and in the report written by Licznar and Mikołajewski (2021), also following the methodology of preparation of dimensionless hyetographs originally proposed by Huff (1967). Each cumulative hyetograph of heavy rainfall with known total duration was divided into 100 equal time intervals. For each of the subsequent intervals, a corresponding cumulative rainfall increase was determined. Subsequent cumulative rainfall increments were divided by total depth to obtain unitary cumulative rainfall increments. As a result, the shape of each heavy rainfall was modelled by a hyetograph normalised to a range from 0 to 100% for duration and to a range from 0 to 1 (100%) for depth.

Mutual comparison of the shapes of dimensionless hyetographs within the analysed sets of heavy rainfalls from a hundred analysed stations, and further designation of clusters of typical (model) rainfall hyetographs employed the expanded methodology described in detail in paper by Mikołajewski et al. (2022). The applied research methodology covered tools for hierarchical and non-hierarchical analysis of the structure of groups of dimensionless heavy rainfall hyetographs. The application of the hierarchical agglomeration method permitted preparation of dendrograms of similarity of dimensionless hyetographs in the analysed groups (Block [8] in Fig. 1). The dendrograms were subject to expert analysis for the purpose of determination of a potential number of clusters in the analysed groups, and identification of particularly specific rainfalls (Block [9] in Fig. 1).

Specific rainfalls observed in the dendrograms (Block [10] in Fig. 1) (separate rainfalls incompatible with others) were analysed by assessing their course over time (Block [12] in Fig. 1). Example of dendrograms with specific rainfall is presented in Fig. S1 supplementary information. Experts rejected rains whose intensity was greater than the measuring capacity of used rain gauge, or those whose hyetograph suggested that the device was not working properly (Block [13] in Fig. 1). Incorrectly recorded hyetograph, which was caused by clogged funnel, is presented in Fig. S2 in supplementary information.

Then, for corrected groups (with the exception of specific rainfalls), diagrams of dependency of Caliński and Harabasz Index (*CHIndex*) values and total within sum of squares (*wss*) (Mikołajewski et al. 2022) on the number of *k*—clusters were prepared (Block [11] in Fig. 1). The analysis of these diagrams provides the basis for the determination of the cluster number for which a division of clusters of heavy rainfalls should be performed from the point of view of similarity of their dimensionless hyetographs.

The maximum value read from the CHIndex chart combined with the value in which the gradient of the wss index decrease significantly allowed to determine the value of k corresponding to the number of clusters (Block [15] in Fig. 1), (Fig. S3 in supplementary information).

The accuracy of adopting the optimum cluster number (meeting the requirements of internal coherence and external isolation) was verified at the stage of clustering of sets of hyetographs by means of the k-mean method and with the application of bootstrap (with 150 repetitions of the grouping algorithm for each of the sets and number of clusters in the range of 2–20), (Block [16] in Fig. 1). The number 150 was determined based on examining the course of the function of the ratio of standard deviation to the mean for the bootmean values for number of simulations of the Bootstrap parameter in the range of 20–250. For bootstrap = 150, the lowest not exceeding 6% values were obtained. Example is shown in Fig. S4 in supplementary information. The accuracy of the adopted number of clusters and the performed division of hyetographs was verified through control of obtaining the average Jaccard index value for each of the clusters at a minimum level of 0.6 (Mikołajewski et al. 2022). In the case of a lower Jaccard index value for any of the clusters, the number of clusters was reduced, and the bootstrap calculations were repeated until obtaining Jaccard index values not lower than 0.6 for all clusters. After meeting that criterion, centres of gravity were determined for all clusters (arithmetic means calculated from the original values of each variable based on objects included in a given cluster). As a result, averaged cumulative dimensionless hyetographs of heavy rainfalls were obtained for the designated clusters. A set of model hyetographs was eventually identified for each rain gauge (Block [17] in Fig. 1).

In each set of model hyetographs, the frequency of occurrence of rainfalls with hyetographs classified to particular clusters was analysed (Block [18] in Fig. 1). Moreover, profiling of clusters was conducted with the application of variables that did not participate in the process of classification of the set of objects. Such variables included total depths and total durations, as well as the resulting mean intensities of heavy rainfalls.

## RESULTS

### Heavy rainfalls and their characteristics

**Fig. 2:**
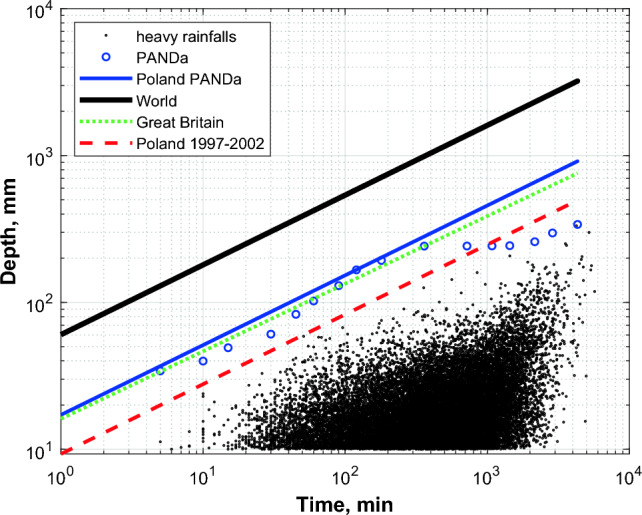
Set of 31 646 designated heavy rainfalls on the background of scale dependencies of maximum rainfalls recorded globally (Paulhus 1965), in Great Britain (Wilson 1990), and in Poland in selected stations during floods in the years 1997–2002 (Banasik 2005, 2009). Moreover, the diagram shows maximum phase rainfalls designated in the scope of the PANDa project on a network of a hundred stations in Poland for durations from 5 to 4320 min with their scale dependency

The numbers of designated heavy rainfalls in a hundred analysed stations are presented in Table S1 in supplementary information. According to the data from Table S1 (Supplementary information), the number of designated heavy rainfalls (except for strongly deviating rainfalls with particularly specific precipitation models) varied from 200 to 726 within 100 analysed rain gauges. Nonetheless, in the case of three stations to reach at least a 200-element sets, it was necessary to supplement the number of heavy rainfalls by events designated from additional observation years. For the rain gauge from Poznań, heavy rainfalls from 2018 and 2019 were added, for the rain gauge from Biebrza from 2017, and for the rain gauge from Chwałkowice from 2016 and 2017. A total of 31 646 heavy rainfalls were therefore designated and accepted for further analyses. The depths and durations of all the designated heavy rainfalls are presented in Fig. 2.

Pursuant to expectations, total amounts of heavy rainfalls did not exceed maximum interval precipitation amounts designated in the implementation of the PANDa project (Licznar et al. 2020), or the values described by the scale dependency of the probable maximum precipitation (PMP) (Block [4] in Fig. 1) (Banasik and Ostrowski 2010):

6 $$P_{D} = 65 \cdot D^{0.475} ,$$

where *P*_*D*_—point probable maximum precipitation (mm), *D*—rainfall duration (h).

However, the scale dependency (6) was determined based on an independent set of heavy rainfalls, collected by Ozga-Zielińska and Ozga-Zieliński (2003), that caused the greatest floods in Poland in the multiannual period 1997–2002.

Values of probable maximum precipitation PMP determined based on formula (6) are approximately 3.5 times lower than probable maximum precipitation PMP determined globally (Paulhus 1965). It is, however, not methodically justified to compare values of precipitation extremes in Poland with precipitation extremes recorded by rain gauges in other climate zones or at considerably different latitudes, i.e. in areas with substantially higher annual precipitation totals. Due to this, Ozga-Zielińska and Ozga-Zieliński (2003) postulated operating on relative values of maximum precipitation, referred to annual normal precipitation. In that case, the relative record precipitation values from Poland become approximate to those of relative global record precipitation with more than day-long durations, e.g. from India or the Philippines. According to Banasik and Ostrowski (2010), relative values of record precipitation from Poland presented by Ozga-Zielińska and Ozga-Zieliński (2003) can be described by a common formula:

7 $${\raise0.7ex\hbox{${P_{D} }$} \!\mathord{\left/ {\vphantom {{P_{D} } {P_{y} }}}\right.\kern-0pt} \!\lower0.7ex\hbox{${P_{y} }$}} = 0.10 \cdot D^{0.40} ,$$

where *P*_*D*_—point probable maximum precipitation PMP (mm), *P*_*y*_—annual normal precipitation (mm), *D*—rainfall duration (h).

**Fig. 3:**
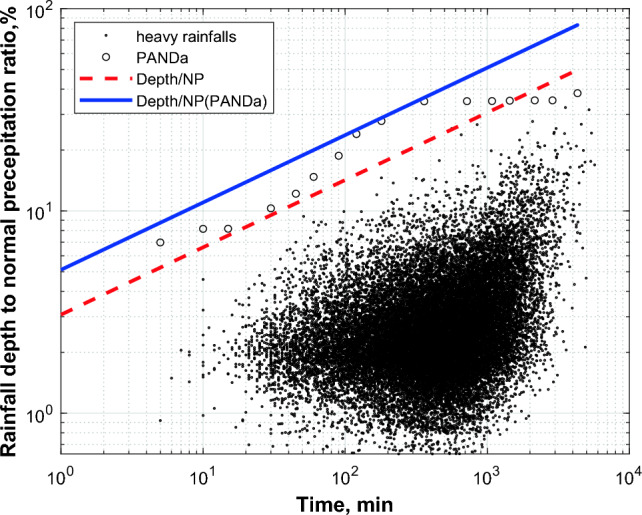
Scatter plot of ratios of depths of 31,646 designated heavy rainfalls to the corresponding normal precipitation in the duration function. Analogical ratios for maximum depths of interval (phase) rainfalls designated in the scope of the PANDa project in a network of a hundred stations in Poland for durations from 5 to 4320 min are also marked on the plot. The upper limiting scale dependencies were determined for both sets

Relative depths of 31 646 designated heavy rainfalls were calculated through dividing their depth by normal precipitation determined for particular stations. The resulting values are plotted versus rainfalls durations on scatter plot in Fig. 3.

The highest of the obtained values exceeded threshold values described by formula (7). Due to this, the red colour (dotted line) in the plot denotes the course of a new, higher scale equation:

8 $${\raise0.7ex\hbox{${P_{D} }$} \!\mathord{\left/ {\vphantom {{P_{D} } {P_{y} }}}\right.\kern-0pt} \!\lower0.7ex\hbox{${P_{y} }$}} = 0.12 \cdot D^{0.40} ,$$

where *P*_*D*_—point probable maximum precipitation PMP (mm), *P*_*y*_—annual normal precipitation (mm), *D*—rainfall duration (h).

It is worth emphasising that although the higher scale dependency limiting maximum relative rainfall depth was drawn, according to the expectations, the curve was below the curve previously developed for relative maximum phase precipitation from the PANDa project. The latter curve is marked in Fig. 3 with blue colour, and is described with the following formula (Licznar et al. 2020):

9 $${\raise0.7ex\hbox{${P_{D} }$} \!\mathord{\left/ {\vphantom {{P_{D} } {P_{y} }}}\right.\kern-0pt} \!\lower0.7ex\hbox{${P_{y} }$}} = 0.20 \cdot D^{0.40} ,$$

where *P*_*D*_—point probable maximum precipitation PMP (mm), *P*_*y*_—annual normal precipitation (mm), *D*—rainfall duration (h).

As already mentioned, in the case of each station, the obtained sets of heavy rainfalls were analysed in terms of distributions of rainfall depths, durations, and mean intensities. For this purpose, histograms of the aforementioned parameters were prepared, and probability distributions were fitted (2-parametric exponential, gamma, and GEV distributions, respectively) (Block [5] in Fig. 1). Example histograms with fitted probability distributions prepared for the rain gauge from Warsaw, the capital of Poland, are presented in supplement as Figs. S5–S7. Moreover, for a combined set of 31 646 designated heavy rainfalls, histograms of rainfall depth, duration, and mean intensities were prepared, and relevant probability distributions were fitted: 2-parametric exponential, gamma, and gev. The histograms with fitted probability distributions are presented in supplementary information—Fig. S8. Except that the variability of parameters of fitted probability distributions within a hundred analysed stations is presented in supplement as Fig. S9. The analysis indicates substantial variability in the distribution parameters fitted to data from 100 meteorological stations analysed in Poland. The box plots illustrate the dispersion of parameter values for different distributions, suggesting differences in climatic conditions among various regions of the country. Moreover, the identification of outliers underscores potential anomalies in the data that could be significant for further climatological analysis.

The investigation of heavy rainfalls also covered the analysis of the frequency of their occurrence in particular stations, as well as mean total rainfall depths and mean total durations. Pursuant to the limitations of the base of the PANDa project, the analysis was narrowed down to the summer hydrological half-year (from 1 May to 31 October). In this period, a total of 28 457 heavy rainfalls occurred the statistics of which are presented in Table S2 (Supplementary information). Table S2 which shows PANDa project stations’ statistics of the designated set of heavy rainfalls of the summer half-year is presented in supplement. According to the data in the table, the mean frequency of occurrence of heavy rainfalls in the summer half-year is 9.5 heavy rainfall events per year, and its highest value of 22.5 heavy rainfall events per year was recorded for Hala Gąsienicowa station located in Tatra Mountains (1523 m a.s.l.). The lowest value of 5.7 heavy rainfall events per year was captured at Poznan station (88 m a.s.l.). The determined frequencies are in accordance with expectations and results of previously published research from the territory of Poland. For example, Licznar et al. (2011) designated 250 heavy rainfalls from a set of digitised precipitation records from pluviographs from 38 years from Wrocław (south-west Poland) using analogical criteria. They determined their frequency of occurrence at a level of 6.6 times per year. For comparison in Warsaw, the capital of Poland, in a network of 25 electronic weight rain gauges recording precipitation all year round, a total of 669 heavy rainfalls were designated by Licznar and Szelag (2014), which was equivalent to a frequency of occurrence of heavy rainfalls equal to 12.3 events per year.

The frequencies of occurrence of heavy rainfalls are evidently higher in the belts of mountain and coastal stations. Analogically, the longest mean annual durations of rainfalls of the summer half-year and mean annual depths of heavy rainfalls of the summer half-year are recorded for mountain stations. The longest mean durations of heavy rainfalls and their highest total depths of 260.2 h and 667.2 mm, respectively, were again observed for the highest located rain gauge on Hala Gąsienicowa. For comparison, mean values of these parameters in the set of 100 analysed stations are 85.4 h and 191.4 mm, respectively.

### Hyetographs of heavy rainfalls and their classification

For all hundred stations, their cumulative, dimensionless (normalised) hyetographs were developed for all designated heavy rainfalls. At the next stage of the study, by means of the methods of hierarchical agglomeration, dendrograms of similarity of temporal courses of rainfalls were prepared in the analysed sets of particular rain gauges. Prior more advanced clustering studies, particularly specific, most probably improperly recorded precipitation patterns, like presented in Fig. S2, were removed (in analogical way like in the case of research from Kraków, Mikołajewski et al. 2022). The necessity of removal of extreme outliers among hyetographs occurred only for 29 heavy rainfalls recorded in 11 out of 100 analysed stations, constituting less than 0.09% of the entire base of heavy rainfalls.

Based on the graph analysis, the optimum and maximum number of clusters *k* was determined using WSS and CHIndex values. The accuracy of the chosen number of clusters and the hyetograph classification was verified through bootstrapping. If any cluster's Jaccard Index was too low, the initial number of clusters k was reduced, and bootstrapping was repeated. Consequently, as detailed in Table S1, the final cluster numbers were set as *k* = 3, *k* = 4, and *k* = 5 for 37, 58, and 5 rain gauges, respectively. In all clusters, the Jaccard Index values were maintained above 0.6, confirming the robustness of the clustering.

**Fig. 4:**
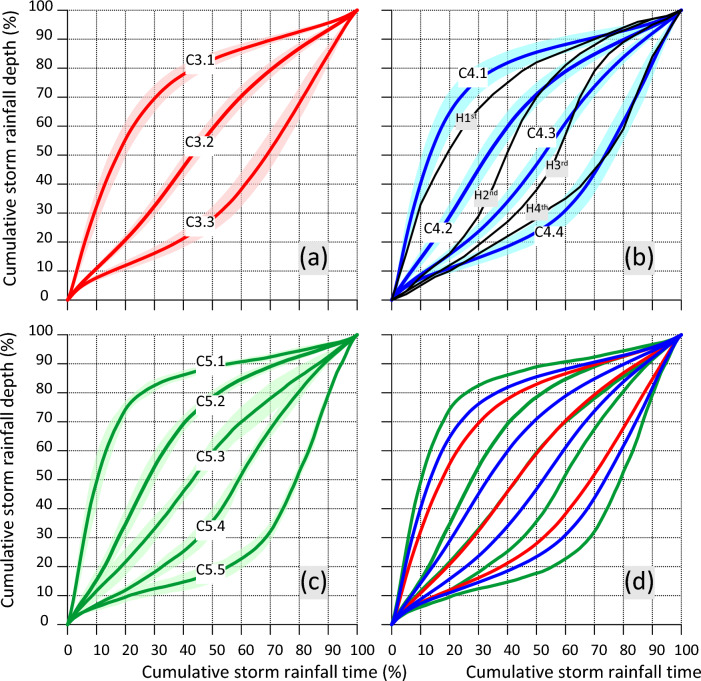
Diagram **a** shows averaged dimensionless cumulative rainfall hyetographs for three clusters identified through k-means clustering using a 37-gauge subset in Poland (optimum cluster number: 3). Diagram **b** displays averaged dimensionless cumulative rainfall hyetographs for four clusters identified using a 58-gauge subset in Poland (optimum cluster number: 4), including a comparison with Huff's Median Time Distributions of Heavy Storm Rainfall at a Point (1990). Diagram **c** presents averaged dimensionless cumulative rainfall hyetographs for five clusters identified using a 5-gauge subset in Poland (optimum cluster number: 5). The mean values for each cluster are represented by lines on a semi-transparent background of standard deviation ribbons. Diagram **d** contains a summary of averaged cumulative precipitation hyetographs for all three subsets of gauge sites

Averaged cumulative dimensionless hyetographs of heavy rainfalls were determined for the designated clusters (Fig. 4). The variability of the resulting courses of model hyetographs in subsets of 37, 58, and 5 stations for which the optimum cluster number was *k* = 3, *k* = 4, and *k* = 5, respectively, is presented on the graphs in Fig. 4. Moreover, frequency of occurrence of heavy rainfalls with a distribution approximate to the determined model hyetographs was calculated for each station. The variability of per cent shares of heavy rainfalls classified to particular clusters in subsets of 37, 58, and 5 stations for which the optimum cluster number was equal to *k* = 3, *k* = 4, and *k* = 5, respectively, is presented in box plots in supplement as Figs. S10–S12. More detailed analysis of Fig. S11 shows that in the case of stations with a cluster number of *k* = 4, heavy rainfalls with hyetographs classified to clusters No. 3 and 2 were recorded the most often (average of 37% and 31%, respectively). Heavy rainfalls with hyetographs with highly variable rainfall intensity values, corresponding with patterns in clusters 1 and 4, were recorded considerably more seldom (on average 15% and 17%, respectively). The designated standard hyetographs on the aggregate chart (Fig. 4d) evenly cover its surface, indicating continuity between the various types of rainfall distributions. The arrangement of hyetographs on the aggregate chart is symmetrical (Fig. 4d). The most distinct (located at the extremes) are the hyetographs for the cluster with the greatest number of types (5), followed by those for clusters with fewer types. Clusters 3.2 and 5.3 are practically identical.

Analogically as in the case of research in the Kraków polygon (Mikołajewski et al. 2022), also research regarding relations of large-scale forcings in the form of, e.g. depth, duration, and mean intensity of rainfall with particular types of local model hyetographs was undertaken. Results of that research are presented in Figs. S13–S15 shown in supplementary information, including diagrams of probability distribution density fitted to sets of values of depth, duration, and mean intensity of heavy rainfalls classified to subsequent clusters in the case of respective subsets: 37, 58, 5 of the analysed rain gauges for which the optimum cluster number was equal to *k* = 3, *k* = 4, and *k* = 5, respectively.

Distributions of rainfall depths and durations could be modelled by means of 2-parameter exponential distribution and gamma distribution respectively, whereas distributions of values of mean intensities of heavy rainfalls in particular clusters could be fitted with the application of a GEV distribution. In the case of each subset of stations, heavy rainfalls classified to clusters No. 1 due to the shapes of their hyetographs are usually characterised by the highest intensities and shortest durations. This is fully confirmed by data presented in Table 1 regarding mean values of depths, durations, and mean intensities of rainfalls classified to particular clusters. For example, values of mean rainfall intensity and duration in cluster No. 1 in a subset of 58 stations with determined cluster number *k* = 4 were 0.092 mm min^−1^ and 356 min, respectively. For comparison, mean intensities and durations of rainfalls in clusters No. 2, 3, and 4 were 0.072, 0.059, and 0.054 mm min^−1^ and 587, 672, and 562 min, respectively. Similar observations can be done for the two remaining subsets of stations. They are analogical to conclusions from research by Huff (1967, 1990) in the USA and Mikołajewski et al. (2022) in Kraków. Huff (1990) postulated the application of first-quartile hyetographs for time scales of approximately 6 h and shorter in designing and modelling drainage systems, and second-quartile heavy hyetographs for time scales from approximately 6–12 h.

**Table 1:** Mean values of total depths, durations, and intensities of heavy rainfalls classified to particular clusters

Cluster	Mean rainfall depth, mm	Mean rainfall duration, min	Mean rainfall intensity, mm min^−1^	Number of rainfalls	% of all rainfalls
37-gauge subset with cluster number *k* = 3					
Cluster No. 1	19.00	405.8	0.0896	2883	9
Cluster No. 2	20.28	651.7	0.0626	5720	18
Cluster No. 3	19.07	588.5	0.0572	3140	10
58-gauge subset with cluster number *k* = 4					
Cluster No. 1	18.90	355.7	0.0919	2784	9
Cluster No. 2	20.14	587.0	0.0724	5729	18
Cluster No. 3	20.92	672.0	0.0587	6743	21
Cluster No. 4	18.65	562.2	0.0541	3032	10
5-gauge subset with cluster number *k* = 5					
Cluster No. 1	18.00	348.0	0.0807	168	1
Cluster No. 2	18.79	576.4	0.0746	409	1
Cluster No. 3	19.49	769.4	0.0504	518	2
Cluster No. 4	20.07	798.8	0.0459	409	1
Cluster No. 5	17.65	557.4	0.0456	168	1

### Regionalization of sets of model hyetographs and spatial variability of heavy rainfalls

A broader analysis of the spatial variability of the heavy rainfall regime in Poland will be included in a separate study. This subchapter presents selected and simplified results, important in the context of hyetographs modelling.

**Fig. 5:**
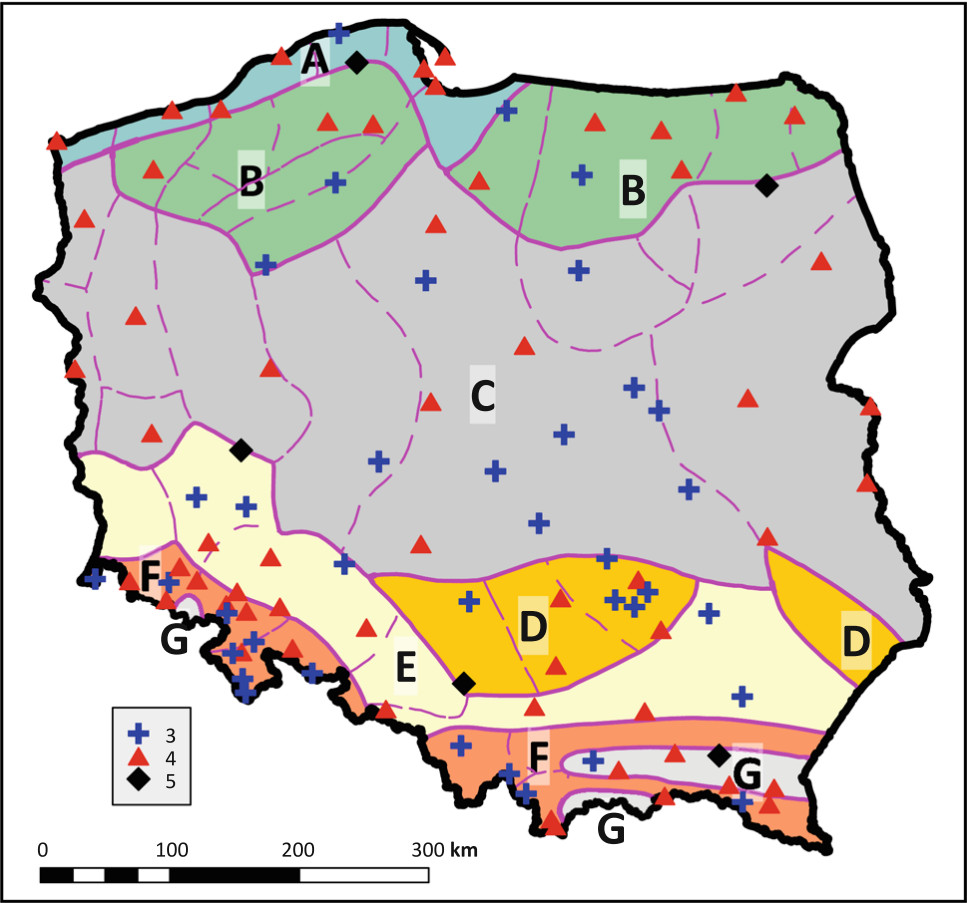
Determined cluster numbers of model hyetographs on a grid of 100 analysed rain gauges in Poland on the background of climatic regions of Poland (Romer 1949). Explanations: A—Baltic climates, B—lakeland climates, C—climates of the “Land of Great Valleys”, D—climates of central uplands, E—climates of submontane lowlands and basins, F—mountain and submontane climates, G—mountain basin climate. Dotted lines are boundaries of lower order regional units

Climatic conditions in the territory of Poland, including precipitation regime, are strongly dependent on two mesoscale factors. One of them is location between the “peninsula” of West Europe and the core of the Eurasian “continent” where there is frequent and active air flow from the west and relatively fast movement of successive pressure systems. Climate in the territory of Poland therefore shows collision of moist air masses from the Atlantic with drier continental masses. This contributes to high year-to-year variability of the spatial and temporal distribution of precipitation, including proportions between solid and liquid precipitation. On the other hand, it contributes to a generally longitudinal course of multiannual means of many climatic parameters. The other factor affecting the characteristics of Polish climate at the mesoscale is the character and genesis of the land relief. The majority, i.e. more than 75% of the territory of Poland, is occupied by low (0–200 m a.s.l.) and relatively flat plain areas, primarily covered with relatively uniform glacial deposits, and mostly used for agriculture. They constitute the northern and central part of the country. In the south, a bipartite zone of uplands, basins, and medium-height mountains occurs, dissected by the depression of the Moravian Gate. Due to the size of Poland and the aforementioned factors, its climatic variability is low, and regional boundaries are vague. In addition to the evident boundary between the mountains with uplands and lowlands, most climatologists also designate a narrow belt of coastal lowlands with a width of several tens of kilometres at the Baltic coast, and a zone of lakelands of north Poland with a high share of postglacial lakes and forests. An example of such regionalisation is the classic study by Romer (1949) (Fig. 5).

**Fig. 6:**
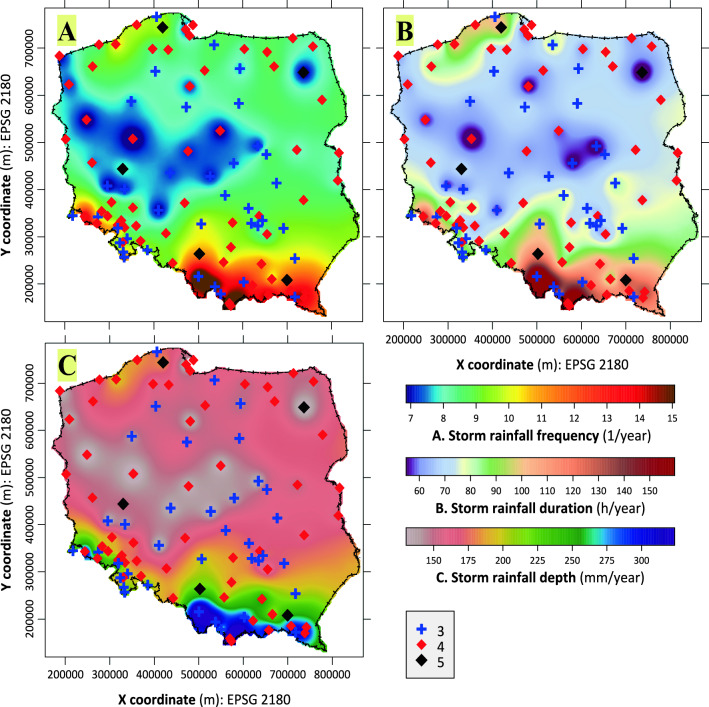
Interpolated spatial distributions of average long-term characteristics of heavy precipitation in the summer half-year. Explanations: **A** frequency of occurrence of heavy rainfalls (1/year), **B** mean duration of heavy rainfalls (h/year), **C** mean depth (total) of heavy rainfalls (mm/year). For better contrast, the colour scale covers a range from 1 to 99% of the variable distribution. The maps also include markings of the locations of sites for which 3, 4, or 5 model hyetographs of heavy rainfall have been established

Statistical tests (Block [26] in Fig. 1) performed for both means (Kruskal–Wallis) and variances (Bartlett) have shown that belonging to clusters with 3, 4, and 5 model hyetographs does not significantly differentiate, in Poland, both due to the average annual frequency of heavy rainfall, as well as their duration and depth (Supplement information, Fig. S16). Also, the geographic distribution of sites belonging to individual clusters (Fig. 6) does not show any relationship with the analysed, annual average, values of parameters that characterise heavy storms. These variables are strongly correlated with each other. For this reason, their spatial distribution is very similar (Fig. 6 and Block [25] in Fig. 1).

It reflects the belt, latitudinal layout of the main relief zones of Poland (the Baltic coast, lake districts created during the last glaciation, a belt of great old glacial valleys and lowlands, and the bidivided—Sudetes and Carpathians—zone of highlands and mountains) and the dominant influence of the north-western circulation from the Atlantic, the North Sea, and the Baltic Sea (Olechnowicz-Bobrowska 1970; Paszyński and Niedźwiedź, 1999; Kirschenstein and Baranowski 2005). Their arrangement is very similar to the total precipitation sums from the summer half-year (May–October) or summer (June–August), regardless of the analysed multi-year period (Olechnowicz-Bobrowska 1970; Paszyński and Niedźwiedź, 1999; Kirschenstein and Baranowski 2005; Lorenc 2005; Łupikasza and Małarzewski 2021) and despite the existence of long-term trends of some characteristics of extreme precipitation (Lupikasza 2010; Pińskwar et al. 2019). This proves the existence in Poland of relatively stable, regional relations within the precipitation field.

The map in Fig. 5 (Block [20] in Fig. 1) visualises lack of evident relations between climatic variability in the territory of Poland and distribution of sites classified to particular clusters due to the number of designated model hyetographs. None of the first level regions is uniform in these terms—each of them includes at least two clusters. Second level regions usually cover an insufficient number of measurement sites (in some cases none) for recognising the uniformity encountered in some cases as credible. However, when approach at the supraregional scale with excluded single outlier locations, the obtained results show certain geographic patterns in their distribution. In the north, in the zone covering coastal lowlands and the northern part of the lakelands (region A and part of region B), a cluster with four model hyetographs is dominant. The situation is similar in the eastern and western part of the belt of “Great Valleys” (region C). In the central part of the belt of “Great Valleys” and the neighbouring southern fragments of the region of lakelands (region B) and uplands (region D), the dominant locations are from a cluster composed of three model hyetographs. Over the remaining area of the “mosaic” of uplands, mountains, and mountain basins (regions D, E, F, and G), sites classified to various clusters neighbour on each other over very small distances. The characteristic location of points classified to a cluster with five model hyetographs is very interesting (Fig. 5). They are located directly at boundaries between first order regions (A/B, B/C, C/E, D/E, and F/G). It is difficult to presume it is incidental.

The distribution of points classified to particular clusters was generalised for the entire territory of Poland by means of Indicator Kriging for qualitative data (Goovaerts 1997; Remy et al. 2009). The method assumes that the applied classification is complete, and the association with classes (groups, clusters) is mutually exclusive (total probability of membership in all classes is 1). Interesting results were obtained in the determination of the surface area occupied by the value of probability of membership in particular “clusters” higher than 0.5. The data considerably differ from the proportions of measurement points classified to them. For a cluster containing three model hyetographs it is only 11.41% (previously 37% of points), for a cluster composed of four hyetographs it is as much as 78.87% (previously 58% points), and for a cluster composed of 5 hyetographs it is only 0.75% (previously 5%). The remaining 8.97% is an area where the probability of membership to any cluster is not higher than 0.5—i.e. the “uncertain” area.

Conclusions resulting from the analysis of the map presented in Fig. S17 in supplementary information are evident. Firstly, they suggest that locations where the optimum division resulted in five model hyetographs are anomalies. Their occurrence is probably related to very local orographic conditions or land cover/use, or specific atmospheric pollutants affecting the stability of the atmosphere and conditions of precipitation development. Secondly, to a certain degree also areas with the occurrence of three model hyetographs can be designated, particularly in north Poland. Three “regions” are clearly marked, however, with a greater range, namely the Kłodzko Basin, western part of the Carpathians (West Beskids), and the belt extending from Rzeszów through Kielce, Łódź, then turning east towards Warsaw. The occurrence of the third, largest zone can be related to the location of the edge of the central Polish uplands and depressions of the Sandomierz Basin and Vistula River valley, although this explanation is currently a hypothesis requiring further verification. Thirdly, it may be suggested that model with four hyetographs is “typical” of the territory of Poland, and that division should be recognised as “default:” in practical applications.

### Changes in the heavy rainfall regime over time

The analysed 30-year period, 1986–2015, is a time of acceleration of climate change (Gulev et al. 2021; Forster et al. 2023). During its duration, previous global air temperature records were exceeded several times (Alexander et al. 2006; Papalexiou et al. 2018; Seneviratne et al. 2021). Precipitation extremes were also observed at that time, although they were continental or regional, rather than global (Alexander et al. 2006; Asadieh and Krakauer 2015; Seneviratne et al. 2021). Therefore, it would be reasonable to check whether there are any time trends in the analysed set of heavy rainfall and whether they could have influenced the results of the classification of their time courses (hyetograms). So far, researchers studying changes in the rainfall regime in Poland (Lupikasza 2010; Pińskwar et al. 2019; Łupikasza and Małarzewski 2021) have not had such comprehensive data on the characteristics of heavy rainfall as used in this study. Since the issue mentioned here is a side topic in this study, the analysis of time variability was carried out in the most simplified way. A more thorough assessment will be carried out in a separate study.

The simplification involved: (1) omitting local and regional variability by aggregating all data from Poland, and (2) assessing temporal variability over three decades (1986–1995, 1996–2005, 2006–2015) and six pentads (1986–1990, 1991–1995, 1996–2000, 2001–2005, 2006–2010, 2011–2015). The first stage of the analysis utilised one-way ANOVA, followed by pairwise comparison tests when the ANOVA indicated a rejection of the null hypothesis (Block [24] in Fig. 1). The ANOVA results for the number of heavy rainfall events, their duration, depth, and intensity divided into decades showed very significant differences for all analysed parameters. The probability that they were accidental was negligible (*p* < 0.00001). The average annual number of torrential rainfall events recorded at the measuring station in the following decades was 8.0, 9.6, and 13.8, average duration: 478.3, 519.3, and 682.8 min, average depth: 19.0, 20.3, and 20.0 mm, and average intensity: 0.072, 0.074, and 0.060 mm min^−1^ (Fig. S18 in supplementary information). This shows that the number and duration of heavy rains increased over a decade, while their depth and intensity increased in the second decade, and either remained at the same level (depth) or decreased significantly (intensity) in the third decade. Pairwise comparison tests showed that while their decadal means differ significantly in the number of rainfall events and duration in each combination, in the case of their depth there is no significant difference between the second and third decades, and in the case of intensity—between the first and second decades. Since the numbers and variances of values in the compared groups differed significantly, Welch's F test and Kruskal–Wallis test were also calculated. The results obtained were fully consistent with those obtained from classic ANOVA.

Since the PANDa database, from which the set of heavy rains analysed in this study was selected, was created by combining registrations from classic analogue pluviographs and automatic tipping bucket rain gauges, it is possible that the observed differences in the rainfall regime in different periods are apparent and are in fact the result of a change in methodology. Until 1998, all measurement stations carried out measurements with analogue pluviographs, and after 2009—only with electronical ones. However, in most locations, the change of recorders took place between 2001 and 2005. If the replacement of measuring devices influenced the statistics of heavy rainfall parameters, the analysis of variance carried out for the pentad periods should show a significant difference between the first three pentads (1986–1990; 1991–1995 and 1996–2000), and the last two (2006–2010 and 2010–2015) with the pentad of 2001–2005 as a transitional one.

An ANOVA performed on the data divided into pentad subsets also showed very significant differences (*p* < 0.00001). The temporal relations, however, are more complicated than before (Fig. S19 in supplementary information). The average annual number of heavy rain events remained at a similar level in the first four pentads (8.5, 7.2, 9.0, and 8.8) and then increased dramatically (13.1 and 14.0). Their average duration in the first two pentads was almost identical (476.2 and 480.9 min). Later, there is a statistically significant, although small, increase: in 1996–2000 and 2001–2005 it was 514.7 and 524.1 min, respectively. In the last two pentads, the duration of heavy rainfall is also similar and significantly longer than before: 691.2 and 674.8 min. The average rainfall depth increases in the first three pentads (18.6, 19.5 and 20.3 mm), then remains at a similar level (20.1 and 20.2 mm) and finally decreases slightly (19.8 mm). The intensity of rain increases slightly at the beginning (0.070 and 0.074 mm min^−1^), then remains at a similar level (0.073 and 0.075 mm min^−1^), and finally decreases very significantly (0.060 and 0.060 mm min^−1^). Pairwise comparison tests showed significant differences between pentads 1–4 and 5 and 6 for the average number of cases. There are no significant differences within these two groups. With regard to the duration of rainfall, the situation is slightly more complex. Statistically significant differences were found, as before, between pentads 1–4 and 5 and 6, and pentad 2 differs from pentads 3 and 4. The rainfall depth recorded in pentad 1 shows very statistically significant differences in relation to all later periods. The difference between pentads 2 and 3 is borderline significant (*p* = 0.03). Other comparisons for this parameter did not show significant differences. The analysis of changes in rainfall intensity showed a previously existing pattern, namely significant differences between pentads in two periods: 1–4 and 5 and 6, with no significant differences within them. In the light of the results of pairwise comparison tests discussed above and the graph in Fig. S19 in supplementary information, we can basically reject the hypothesis that the change of recorders is the reason for the observed trends in the heavy rainfall regime. Pentad 4 (2001–2005), when changes were made to the instrumentation at most measurement stations, is not of a “transitional” nature at all. It is either very similar to the earlier period (number of cases, duration, and intensity) or the later period (depth).

The Chi-square test was used to assess whether the frequency of occurrence of the distinguished types of heavy rainfall time courses changed significantly during the analysed 30-year period (Block [19] in Fig. 1). As before, the entire dataset was divided into decades and pentads. The test was performed on the basis of a summary table, the columns of which represented periods (the entire dataset and decades or pentads), and the rows represented hyetograph types (3, 4, or 5 depending on the cluster). The frequencies of their occurrence in subsequent periods are recorded in individual cells of this table. The obtained results do not indicate the possibility of temporal changes in the frequency of the distinguished types of rainfall time courses. Only in one out of six tests significant differences were obtained (*p* < 0.0001). This was the case when the division into decades was analysed in a cluster of sites with three reference hyetographs. The analysis of the test components showed that the result was caused by a large overrepresentation of type three in the decade 2006–2015, with a simultaneous deficit in the years 1996–2005. This case should be considered random in the context of the remaining results.

## DISCUSSION

Despite being conducted in Poland, a relatively compact area (322 575 km^2^) with a moderate Central European climate, the study found significant spatial variations in the frequency and characteristics of heavy rainfall events. Using a dataset spanning 30 years from a network of 100 rain gauges, the study identified 31 646 heavy rainfall events, mostly (28 457 events) during the summer half-year. On average, there were 10.5 events per year, though excluding extreme values such as those at high mountain stations like Hala Gąsienicowa (22.5 events per year), local frequencies ranged widely from approximately 7 to 15 events annually. Similarly, average durations of heavy rainfall varied widely, from under 60 to around 160 h per year, as did annual depths, from under 150 to over 300 mm per year. Maps developed from this data depict clear patterns akin to longstanding knowledge of precipitation regimes in Poland, influenced by northwest circulation from the Atlantic, North Sea, and Baltic Sea. Annual sums of heavy rainfall generally correlate with total precipitation sums, contributing approximately 30% of annual precipitation at most stations. Comparable studies in Europe, such as those by Madsen et al. (2014), have also noted diverse durations and depths of extreme rainfalls, linked to regional climate patterns and geographic factors.

The analysis found that rainfall depths, durations, and mean intensities at stations could be modelled using the 2-parametric exponential distribution, gamma distribution, and generalised extreme value (GEV) distribution, respectively. Due to spatial variations in the parameters of heavy rainfall events, significant variability was observed in local distributions of rainfall depths, durations, and mean intensities. For instance, parameters for the 2-parametric exponential distribution of rainfall depth across 31 646 heavy rainfalls in Poland were *λ* = 9.819 and *γ* = 10.00, with *λ* ranging from 7 to 13. Similar variability was noted for gamma distributions of rainfall durations, ranging from below 1.4 to close to 2.0 for parameters a, and from slightly above 200 to around 500 for parameters b, after excluding outliers. Overall, these variations in parameters translated into wide ranges for local parameters of mean rainfall intensities (*μ*, *σ*, and *k*), which ranged from slightly above 0.02 to just under 0.04, from 0.016 to 0.025, and from slightly below 0.5 to above 0.8, respectively. For comparison, parameters for mean rainfall intensities across the dataset were *μ* = 0.0295, *σ* = 0.0209, and *k* = 0.6101. These findings underscore the importance of considering local characteristics when analysing extreme hydrological events. Future studies will explore whether parameters exhibit spatial autocorrelation among the 100 stations, potentially enabling spatial estimation for Poland as a whole.

The analysis of heavy rainfall events using high-temporal-resolution data from 1986 to 2015 did not find precipitation maxima exceeding previously established probable maximum precipitation (PMP) values for Poland by Banasik (2005, 2009), based on a limited dataset from major floods in 1997–2002. PMP values from this formula are approximately 3.5 times lower than those from the globally proposed PMP relationship by Paulhus (1965), reflecting Central Europe's precipitation realities under a moderate climate dominated by lowlands. Relative values of maximum precipitation normalised by annual normal precipitation (*P*_*D*_/*P*_*y*_) required a 20% correction based on rainfall durations (formulas (8) and (7)), due to broader database analysis and improved temporal resolution. The relationship between PMP and (*P*_*D*_/*P*_*y*_) from the 31 646 identified heavy rainfall events was lower than relationships from 100 stations analysed under the PANDa project, which focussed on peak maxima. This difference highlights varying rainfall intensities over time during heavy rainfall events.

The literature underscores the importance of capturing rainfall dynamics, specifically momentary intensity variability during events, when studying extreme hydrological phenomena, especially in modelling urban catchments prone to flooding or stormwater retention reservoirs. A significant part of the research focussed on identifying standard hyetographs across 100 locations and regionalizing results to correlate hyetograph types with rainfall event genesis. These are among the first comprehensive studies of this scale in Central Europe, employing modern cluster analysis and geostatistical methods to analyse data from a network of rain gauges, akin to methods used by Castanho et al. (2019) for classifying rainfall events globally.

Based on analysis of parameters like the wss parameter and CHIndex index, optimal hyetograph clusters were determined: *k* = 3, *k* = 4, and *k* = 5 for 37, 58, and 5 rain gauges analysed, respectively. There were no clear correlations found between Poland's climatic diversity and the distribution of classified sites due to varied hyetograph models within individual clusters. The only consistency was stations with five hyetograph classes located at the borders of primary climatic regions. The spatial distribution of clusters with 3, 4, and 5 standard hyetographs did not exhibit clear geographic patterns (Figs. 5, 6), contrary to established knowledge of precipitation patterns (Fig. 6) in Poland. This discrepancy likely stems from the normalisation of genetically different rainfall types with similar temporal patterns in Central European conditions.

Future studies should consider reversing the approach by first dividing geographically or statistically, then classifying normalised hyetographs within those groups. Preliminary cluster analysis of frequency, duration, and depth of precipitation from 100 locations identified three or four distinct groups. Additionally, a two-dimensional frequency histogram for duration and intensity showed that about 90% of rainfall analysed lasted up to 1400 min with intensities up to 40 mm, forming a distinct peak in the dataset.

In-depth spatial analysis of the variability of the number of standard hyetographs using Indicator Kriging suggests that four model hyetographs are the most typical for Poland, and this division should be considered the default in practical applications. Generally, heavy rainfalls with hyetographs most similar to precipitation with uniform intensity were usually recorded for all stations. Heavy rainfalls with hyetographs exhibiting more variable instantaneous rainfall intensity values were recorded approximately 50% less frequently than hyetographs with temporally smoothed intensities. For most frequent stations with a cluster number *k* = 4, heavy rainfalls with hyetographs ascribed to clusters 3 and 2 accounted for an average of 37% and 31%, respectively, of the total rainfall sets. For the same stations, heavy rainfalls with hyetographs classified to models in clusters 1 and 4, with more variable rainfall intensities, were observed considerably less frequently (accounting for an average of 15% and 17%, respectively, of the total rainfall sets). The obtained standard hyetograph patterns at stations divided into four patterns exhibit a clear similarity to the results of earlier studies by Huff (1990) from the USA area. In this regard, the model hyetographs of cluster 1 constitute a specific analogy towards first-quartile heavy hyetographs, postulated by Huff (1990) in designing and modelling drainage systems for time scales of approximately 6 h and shorter. According to the results, events classified into cluster 1 in Central European conditions had significantly shorter durations, usually from 30 to 100 min, and higher average intensities compared to events classified into other clusters.

In the Anthropocene era and amidst climate change, it is obvious that heavy rainfall events and their characteristics cannot be treated as stationary variables. In this context, the results obtained cannot be compared with previous reference statistics since, to the authors' best knowledge, similar analyses on this spatial and temporal scale and using high-resolution rainfall data have not been conducted for Poland and Central Europe. From this perspective, the present study is intended to serve as a reference for future analyses conducted on newly collected datasets of analogous temporal and spatial resolution of rainfall, both in terms of obtained statistics and the potential application of processing methods for registration datasets. The potential of such an approach is evidenced by the results of sectional, simplified analyses of trends in basic characteristics of heavy rainfall events over decades and pentads in the analysed 30-year period from 1986 to 2015. Their main conclusion is the clear and statistically significant changes, manifested especially by the increase in the number and duration of heavy rains in successive decades. However, these clear trends do not align with trends regarding the depths and mean intensities of heavy rainfall events. It is evident that the depth and intensity increased in the second decade, and either remained at the same level (depth) or decreased significantly (intensity) in the third decade. It must be emphasised, however, that these results have very limited reliability, influenced by the too short period of available high-resolution rainfall data and somewhat resulting from their simplified methodology. It can also be presumed that the observed trends, in the form of reductions in the heights and intensities of heavy rainfall events, may be the result of the non-homogeneity of the dataset itself. It should be remembered that around and after the year 2000, the process of conversion from pluviographs to trough automatic rain gauges began, the inherent and confirmed feature of which in many studies is a tendency to underestimate the intensity of rainfall, especially those classified as heavy. Regardless, the obtained results demonstrate the need for systematic, albeit cyclical, monitoring of basic characteristics of heavy rainfall events. It can be assumed that with the increase in new datasets, such comparisons will also be possible regarding changes in developed sets of standard hyetographs for the first time.

## CONCLUSION

It is essential to emphasise the practical, applicative aspect of the conducted research. In engineering issues such as hydrodynamic modelling of drainage systems and when seeking future rainfall scenarios, computer models should use local information about the heights of heavy rainfall combined with equally precise local knowledge of potential distributions of this rainfall over the assumed duration of its occurrence. Information about the heights of heavy rainfall is already readily available for engineers for many areas in the form of rainfall atlases such as NOAA Atlas 14 in the USA,^2^ KOSTRA in Germany,^3^ or PANDa in Poland.^4^ Reference and global assistance can also be provided to engineers by dependencies such as *Py/Pd* or PMP. In the specific case of Poland, statistical distributions of heights and durations of heavy rainfall can also be developed in research. In a broader context, such data are also possible to obtain from global climate model simulations for future climate change scenarios, albeit with the obvious limitations of their resolution and the possibility of further downscaling. Access to information about maximum rainfall heights can be considered common practice, and the practice of using heights resulting from local statistical models is firmly established in engineers' awareness. The need to operate with truly local and potentially possible scenarios of the distribution of maximum rainfall heights appears much less pronounced in engineering consciousness. This lack results from the frequent absence, in many areas of the world, of access to analyses of standard hyetographs, postulated by Huff (1967). In this perspective, the set of standard hyetographs obtained eliminates these gaps for Poland and presumably neighbouring areas of Central Europe. The compiled results indicate the necessity of operating with complete sets of hyetographs as alternative scenarios of rainfall occurrence and variation in engineering solutions based on this, including perhaps considering the frequency of occurrence of individual rainfall scenarios. Operating with a set of 3, 4, or even 5 hyetographs with known occurrence frequencies in the local dataset of heavy rainfall events is a pragmatic approach, not requiring engineers to perform a series of time-consuming simulations but leaving at least a partial margin for probabilistic interpretation of the obtained results. Therefore, the methodology practically tested on a large dataset of 30-year registrations from 100 rain gauges should be applied in other areas where analyses of standard hyetographs in high temporal resolution have not yet been conducted.

## Supplementary Information

Below is the link to the electronic supplementary material.

Supplementary file1 (PDF 2204 kb)

## Footnotes

1 https://retencja.pl/en/aplications/rainbrain/.

2 https://hdsc.nws.noaa.gov/pfds/.

3 https://www.dwd.de/DE/leistungen/kostra_dwd_rasterwerte/kostra_dwd_rasterwerte.html.

4 https://atlaspanda.pl.
