# Supplementary material for: Heavy rainfalls in Poland and their hyetographs
Source: Ambio. 2024 Sep 16;54(1):86–104. doi: 10.1007/s13280-024-02069-6 (PMC11607261; doi:10.1007/s13280-024-02069-6)
Supplement: Supplementary file 1 — Supplementary file1 (PDF 2204 kb) [file 13280_2024_2069_MOESM1_ESM.pdf]

Ambio

Supplementary Information
This supplementary information has not been peer reviewed.

Title: **Heavy rainfalls in Poland and their hyetographs**

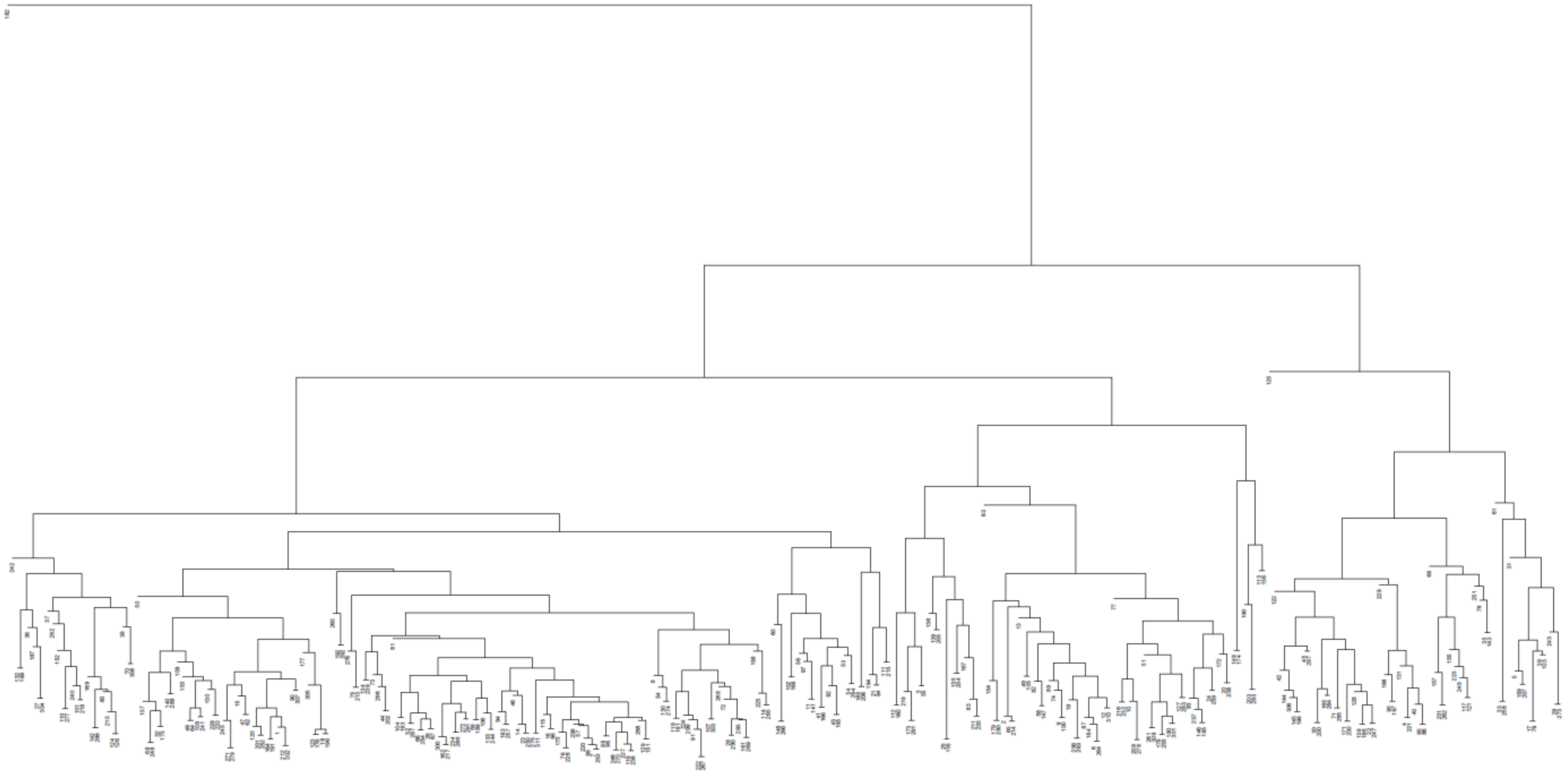

Fig. S1 Dendrogram presenting an example of specific precipitation No. 182 - the top-left branch of the chart, which is dissimilar to any other analysed precipitations in the given location. Such precipitations were excluded during the expert analysis.

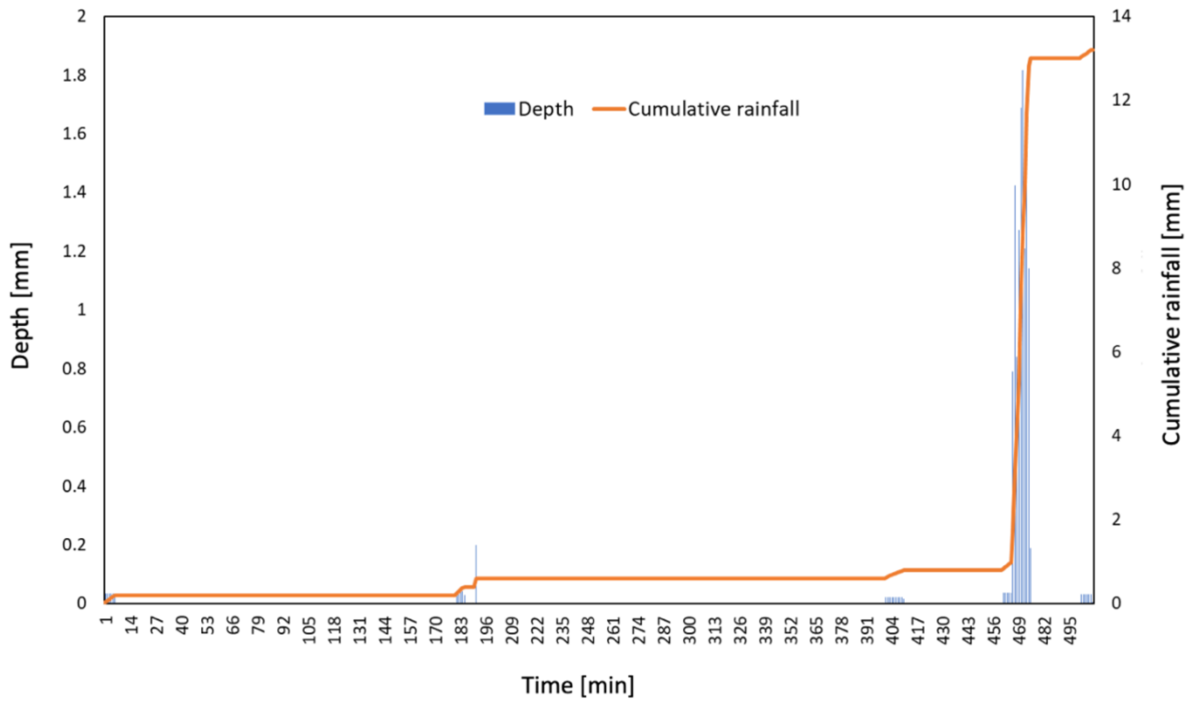

Fig. S2 The chart represents incorrectly recorded precipitation No-182 (same as specific one in Fig. S1). Such a hyetograph is characteristic of a blocked rain gauge funnel.

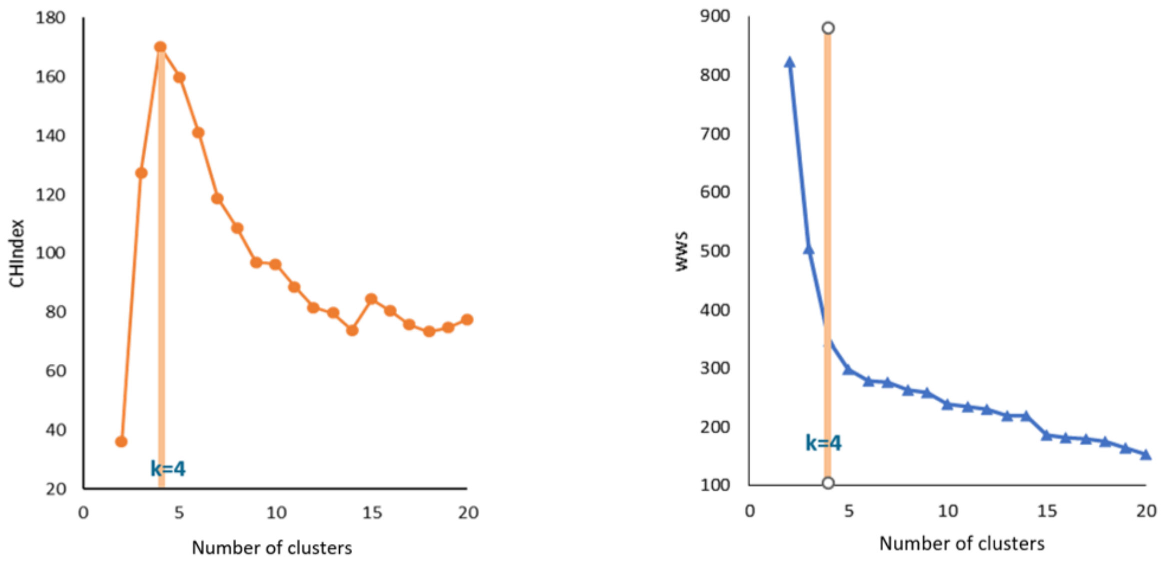

Fig. S3 Charts presenting the example of CHIndex and wss parameters functions, used to assess the optimal number of clusters. In the above example, the maximum of the CHIndex function and the inflection of the wss function were the criteria for separating 4 clusters.

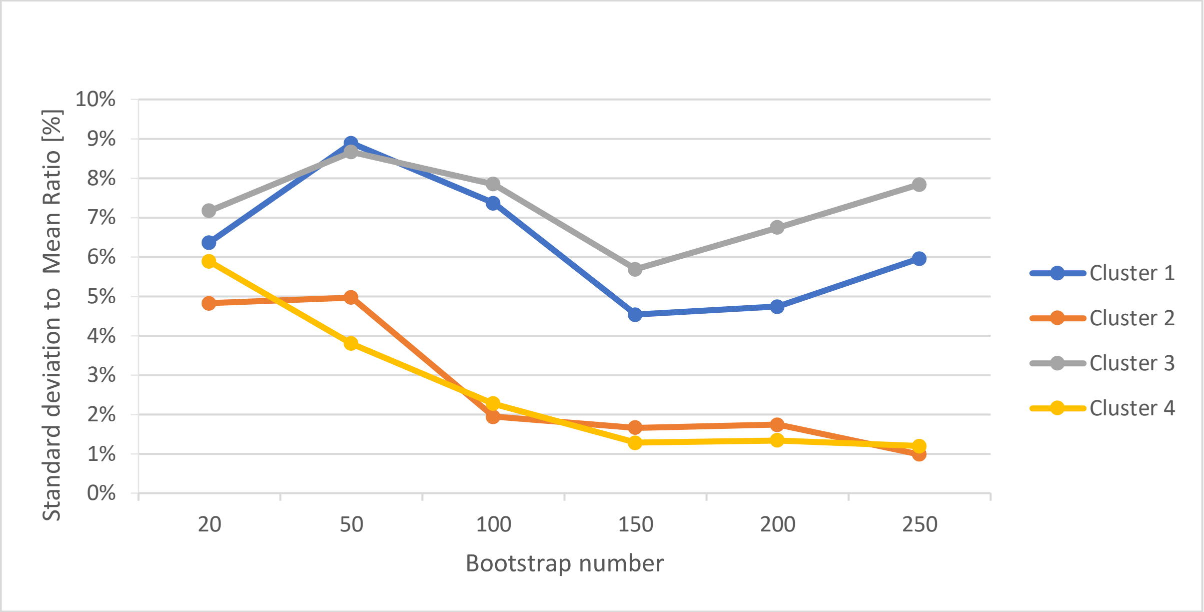

Fig. S4 The chart illustrates the ratio of standard deviation to the mean for the bootmean values calculated for each of the four clusters determined in successive simulations with a bootstrap number of 20, 50, 100, 150, 200, and 250 for a randomly chosen location. The value of 150 resulted in the lowest values of this index, not exceeding 6% for all clusters.

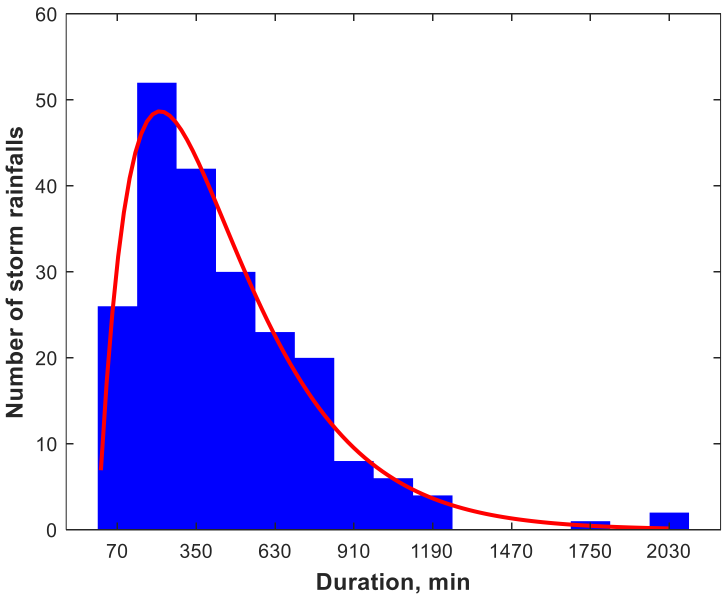

Fig. S5 Histogram of rainfall durations for a set of 214 heavy rainfalls from Warsaw, the capital of Poland, with fitted gamma distribution (*a*= 1,8819, *b*= 239.91).

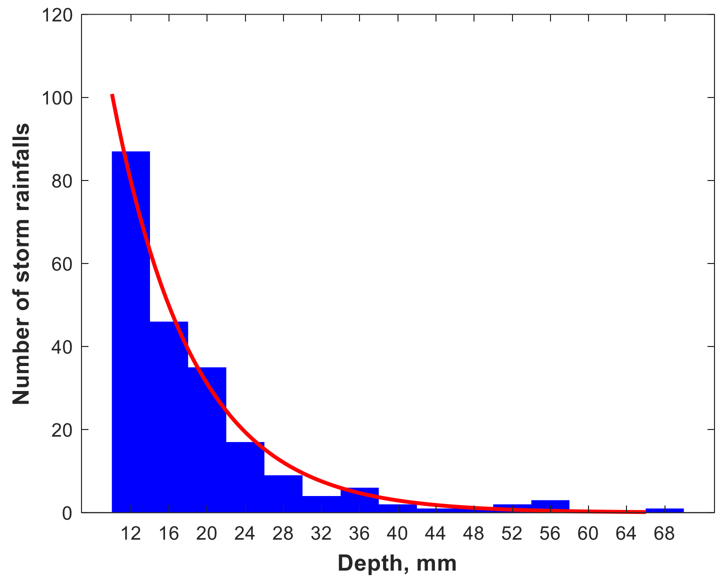

Fig. S6 Histogram of rainfall depth for a set of 214 heavy rainfalls from Warsaw, the capital of Poland, with fitted 2-parameter exponential distribution (λ=0.11794, γ=10,00).

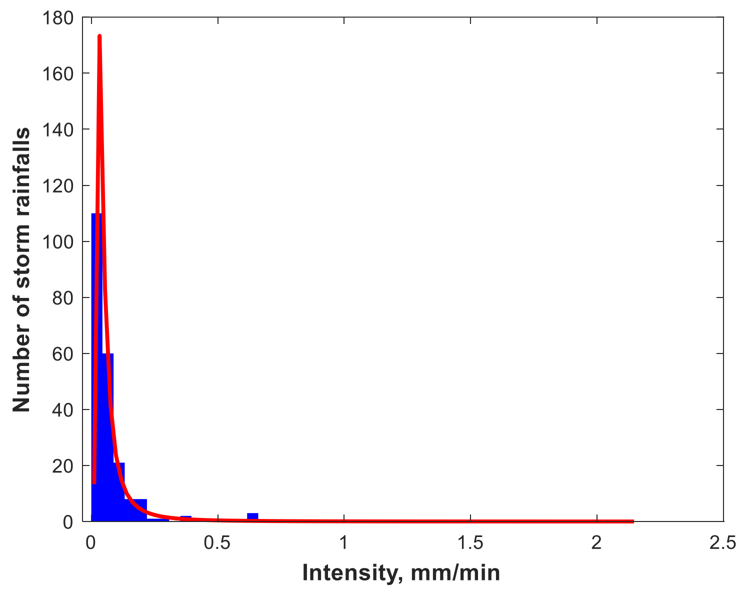

Fig. S7 Histogram of mean rainfall intensities for a set of 214 heavy rainfalls from Warsaw, the capital of Poland, with fitted generalised extreme value distribution (μ=0.03548 σ= 0.02297, k= 0.49621).

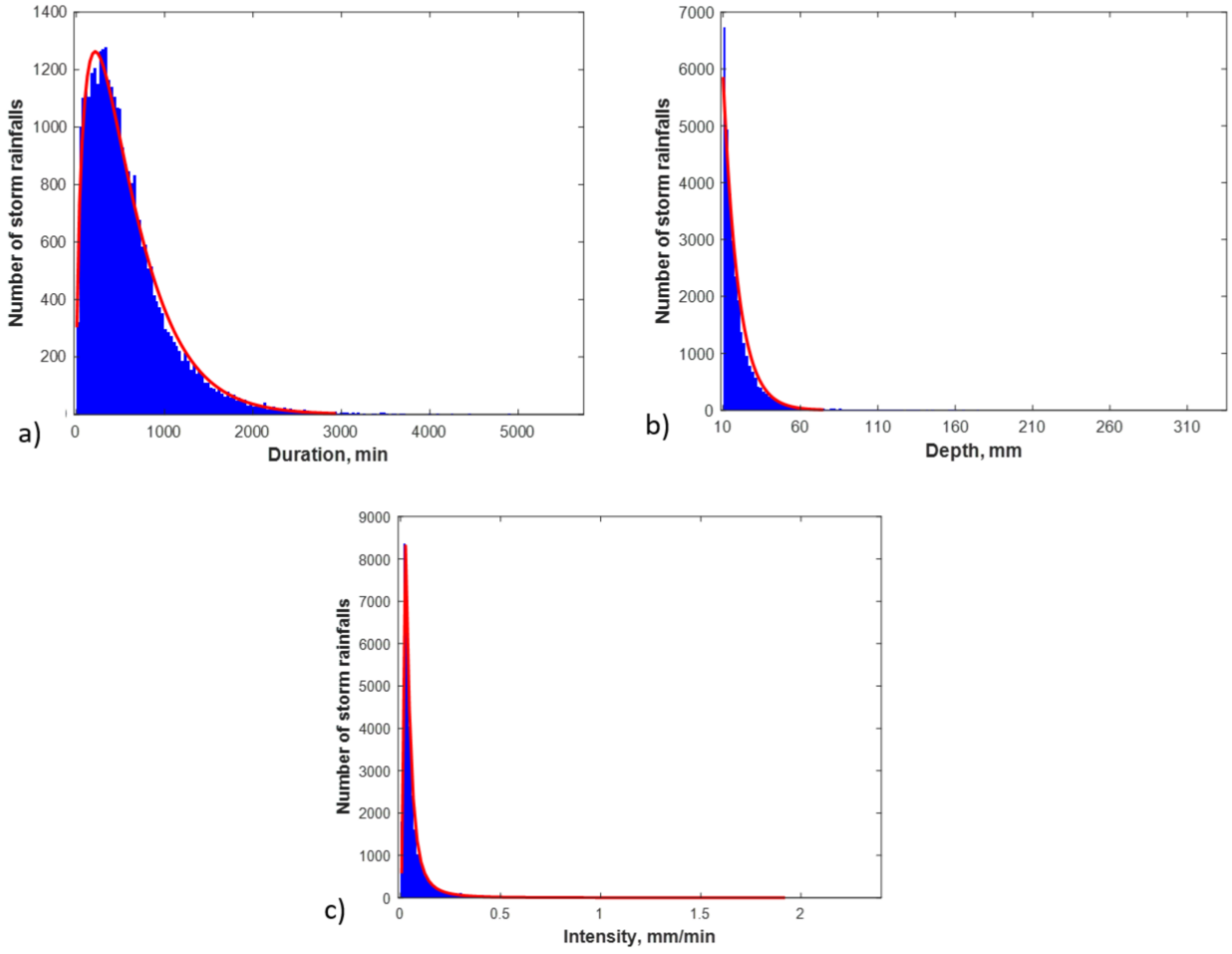

Fig. S8 These diagrams (a, b, c) show rainfall patterns in Poland based on a combined dataset of 31,646 storm rainfalls. Diagram a) displays the histogram of rainfall durations (gamma distribution: a=1,584, b=367.3). Diagram b) shows the histogram of rainfall depths (exponential distribution: λ=9,819, γ=10,00). Diagram c) presents the histogram of mean rainfall intensities (generalized extreme value distribution: μ=0,0295, σ=0,0209, k=0,6101).

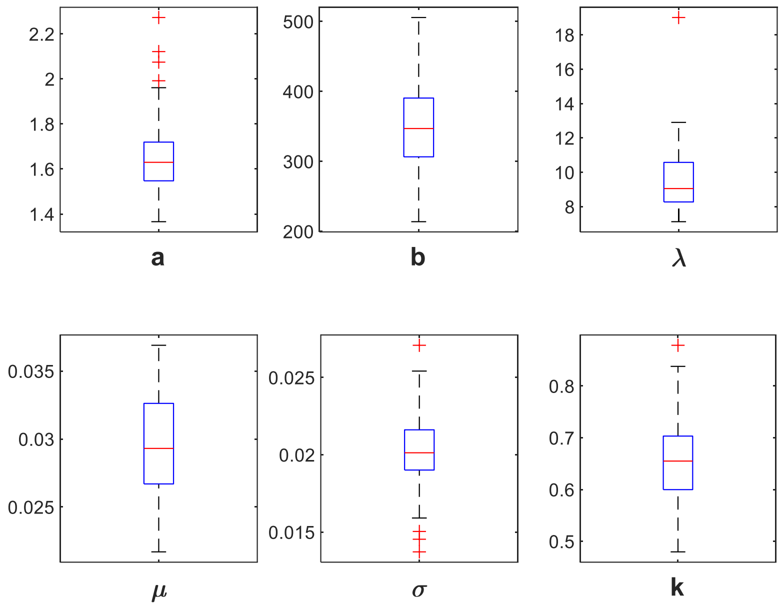

Fig. S9 Box plots showing the variability of fitted distribution parameters among 100 analysed gauges in Poland, respectively for shape a and scale b parameters of gamma distributions, λ parameters of 2-parameter exponential distributions, location μ, scale σ, and shape k parameters of generalized extreme value distributions. The red lines in the middle of each box are samples medians, whereas the bottoms and tops of each box are the 25th and 75th percentiles of the samples, respectively. The whiskers extend to the most extreme data points not considered outliers. Outliers are assumed to be values higher than 1.5 times the interquartile range away from the bottoms or tops of the boxes, plotted individually using the '+' marker symbols.

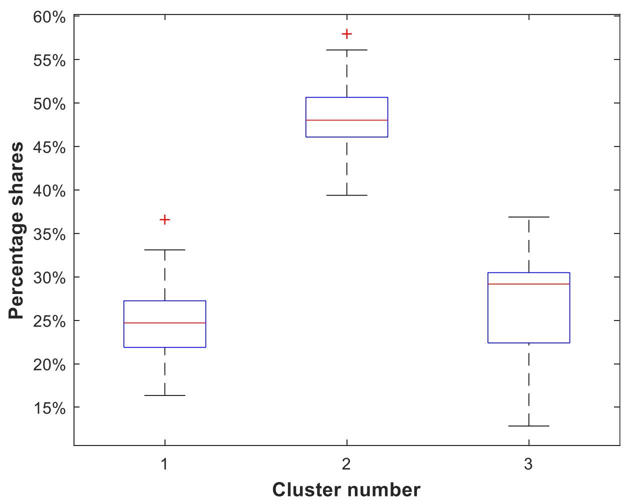

Fig. S10 Box plots showing the variability of percentage shares of rainfalls classified to clusters 1, 2, and 3 among a 37-gauge subset out of 100 analysed gauges in Poland, where the optimum cluster number was equal to 3. Box plots convention is similar to Fig. S9.

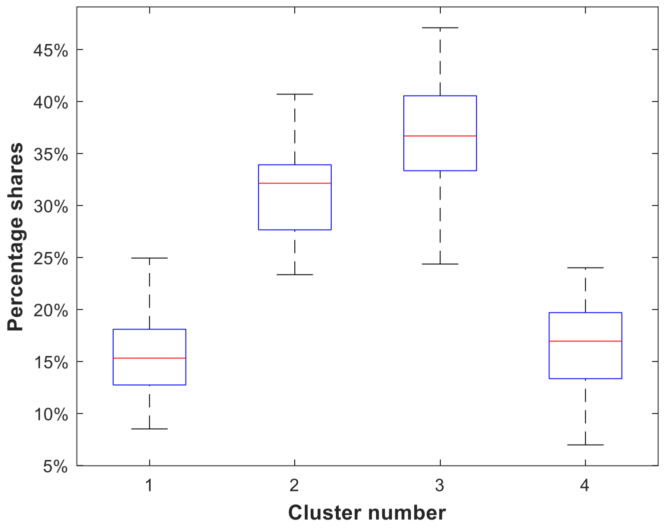

Fig. S11 Box plots showing the variability of percentage shares of rainfalls classified to clusters 1, 2, 3, and 4 among a 58-gauge subset out of 100 analysed gauges in Poland, where the optimum cluster number was equal to 4. Box plots convention is similar to Fig. S9.

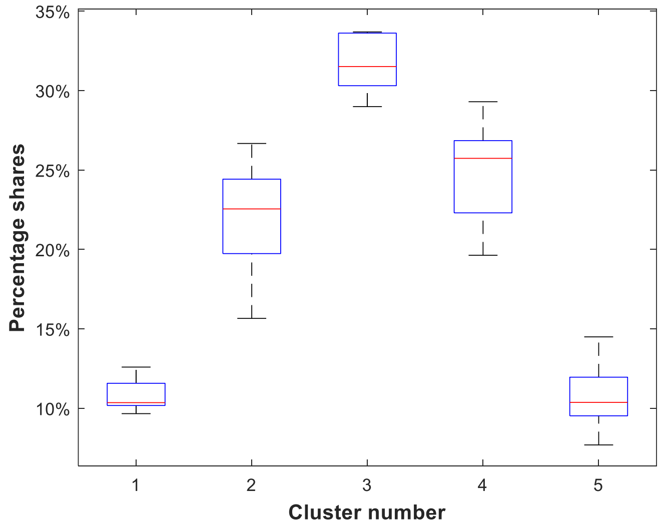

Fig. S12 Box plots showing the variability of percentage shares of rainfalls classified to clusters 1, 2, 3, 4, and 5 among a 5-gauge subset out of 100 analysed gauges in Poland, where the optimum cluster number was equal to 5. Box plots convention is similar to Fig. S9.

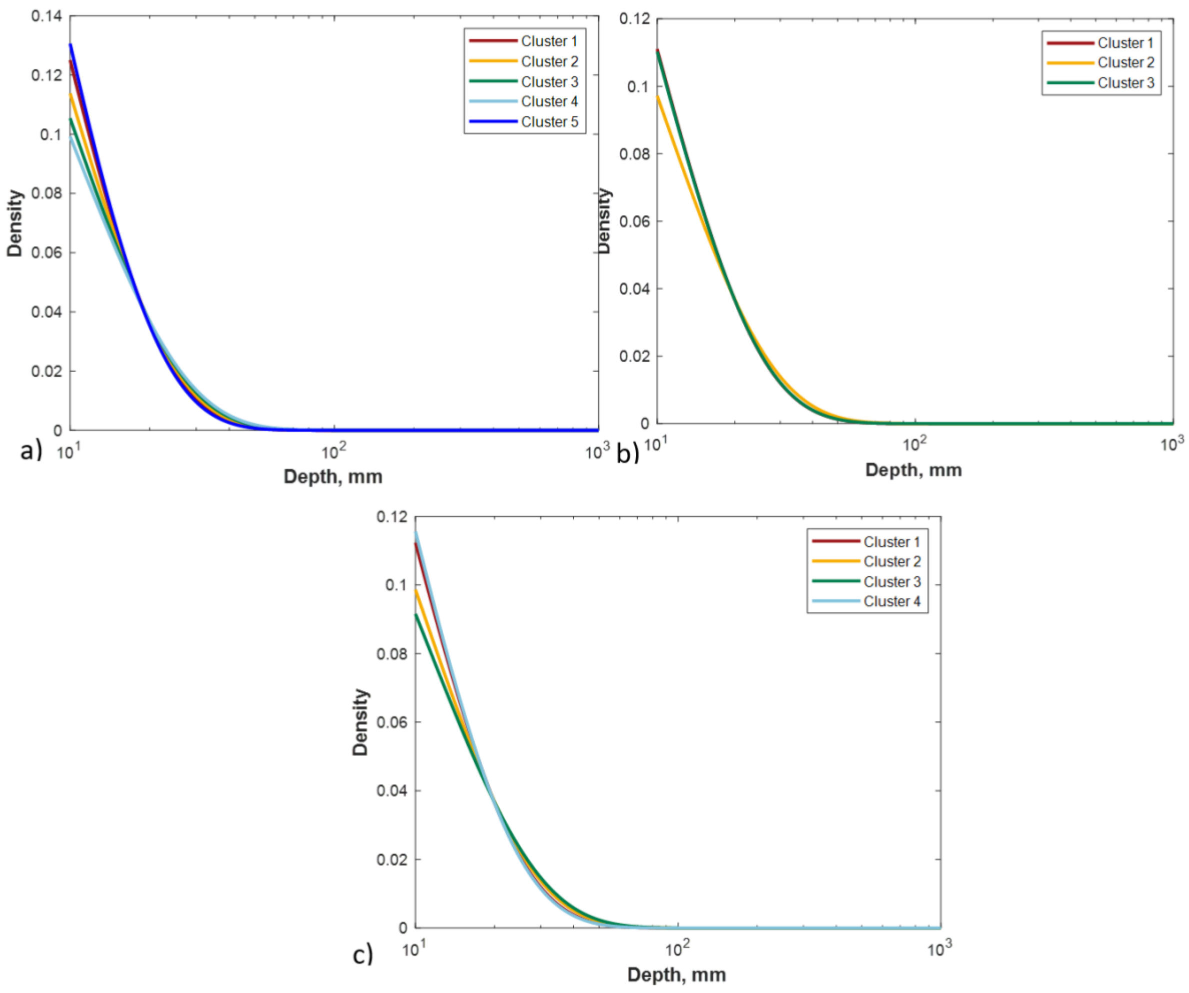

Fig. S13 Diagram a) displays gamma distributions fitted to rainfall depths assigned to clusters 1 to 5 using 5 gauges (optimum cluster number: 5) Diagram b) showcases gamma distributions fitted to rainfall depths assigned to clusters 1 to 4 using 58 gauges (optimum cluster number: 4). Diagram c) presents gamma distributions fitted to rainfall depths assigned to clusters 1 to 3 using 37 gauges (optimum cluster number: 3). All of these distributions are plotted at a log scale for better discrimination and provide insights into the distribution patterns of rainfall depths across different clusters.

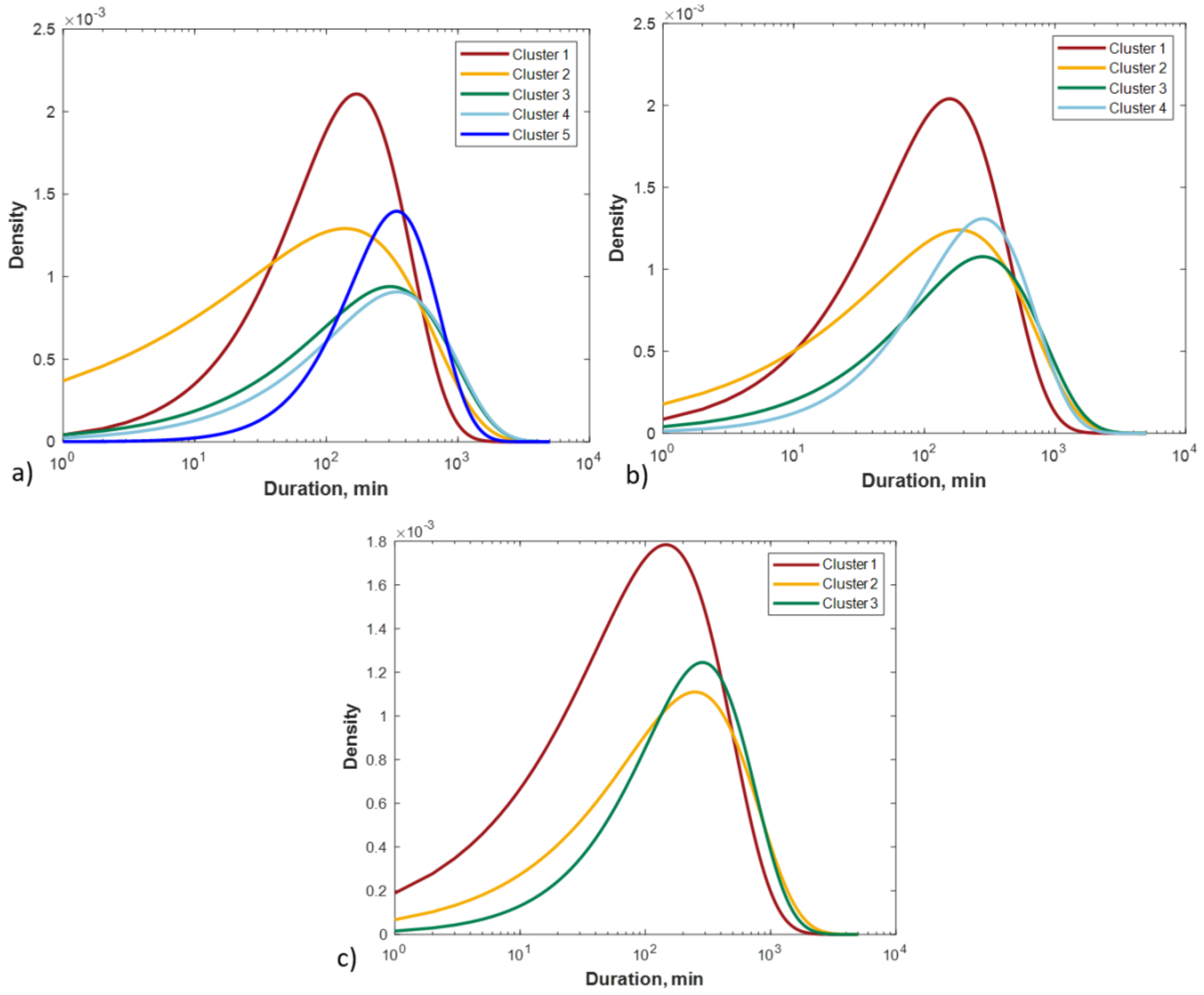

Fig. S14 Diagram a) showcases 2-parameter exponential distributions fitted to rainfall durations assigned to clusters 1-5 using 5 gauges (optimum cluster number: 5). The distributions are plotted on a log scale for improved discrimination. Diagram b) presents the same analysis for clusters 1-4 using 58 gauges (optimum cluster number: 4), also plotted on a log scale. Diagram c) displays the distributions for clusters 1-3 using 37 gauges (optimum cluster number: 3), with a log scale used for better visualization. These figures provide valuable insights into the distribution patterns of rainfall durations across different clusters, visualized using a log scale.

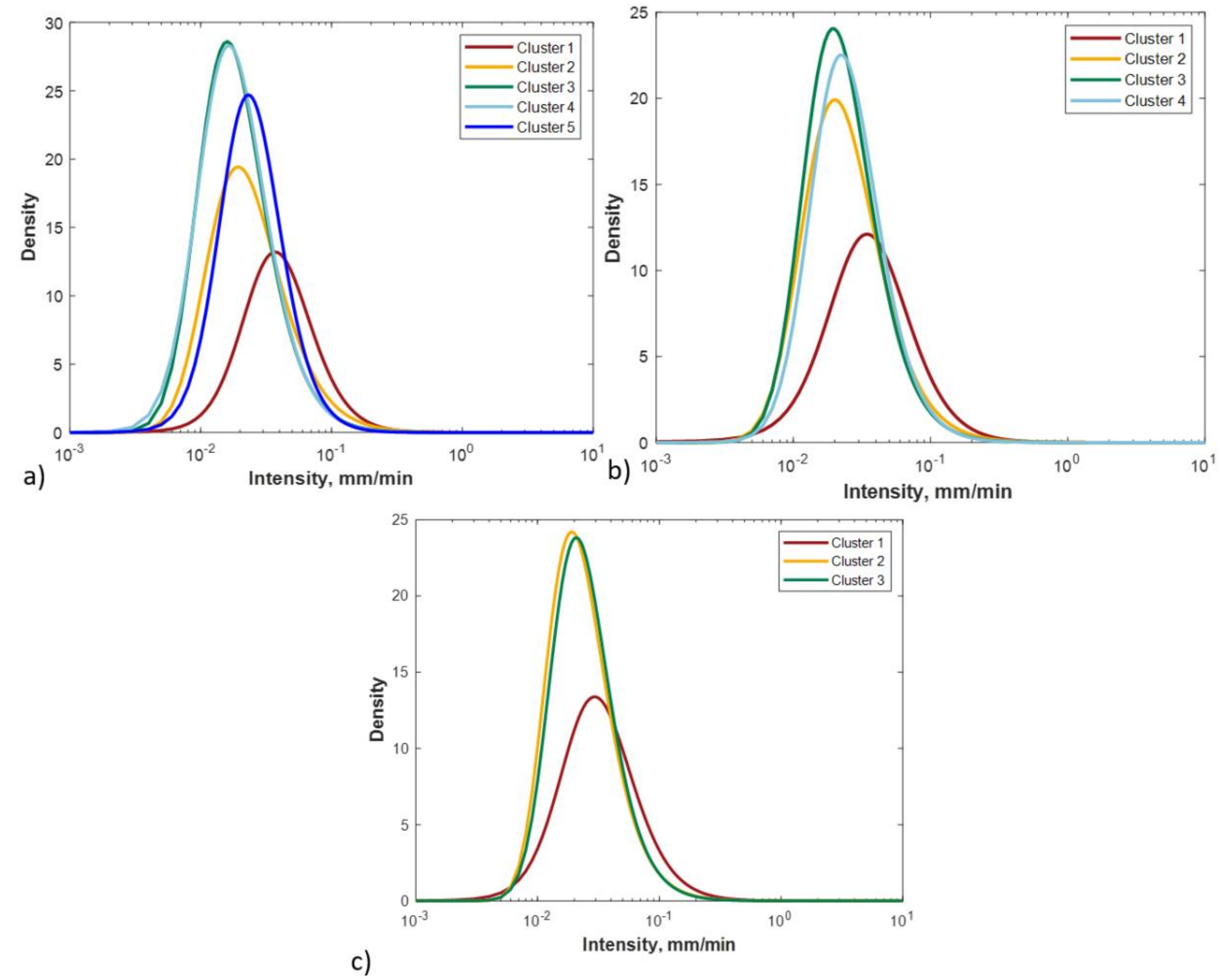

Fig. S15 Diagram a) showcases generalized extreme value distributions fitted to mean intensities of rainfalls assigned to clusters 1-5 using 5 gauges (optimum cluster number: 5). Similarly, diagram b) presents the fitted distributions for clusters 1-4 using 58 gauges (optimum cluster number: 4), while diagram c) displays the fitted distributions for clusters 1-3 using 37 gauges (optimum cluster number: 3). The distributions at all diagrams are plotted with a log scale aiding in enhanced visualization. These diagrams provide insights into the distribution patterns of mean rainfall intensities across different clusters.

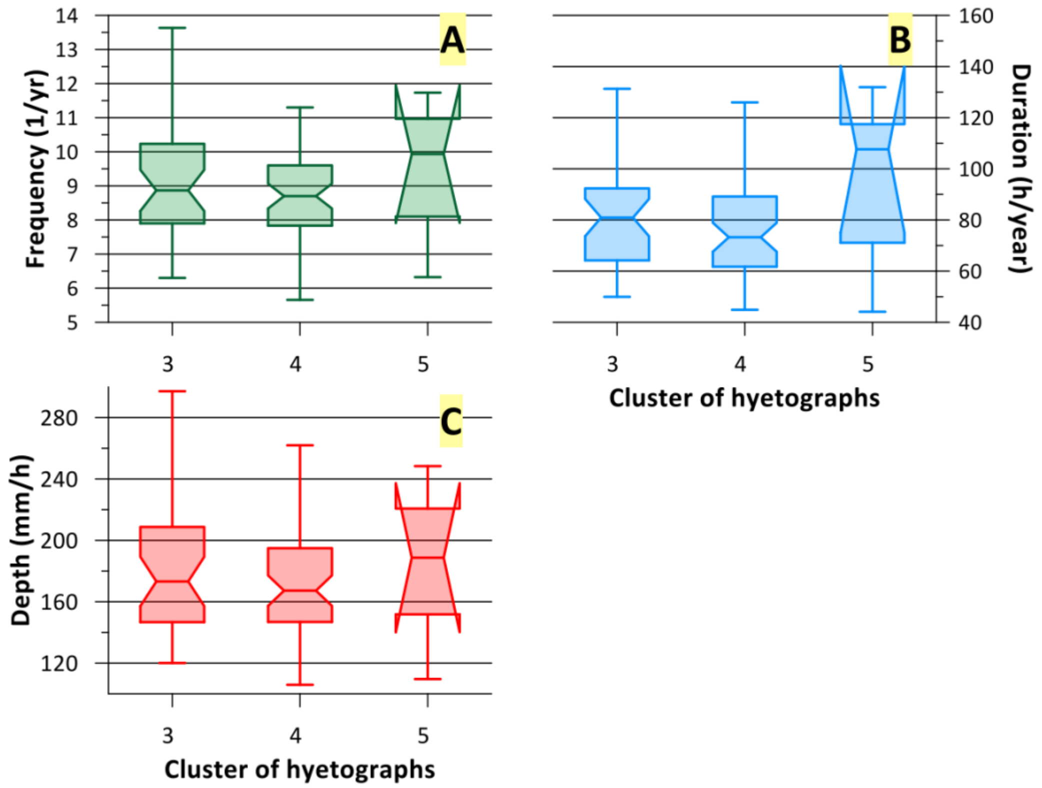

Fig. S16 Boxplots of average multi-year characteristics of storm rainfall in locations of sites for which 3, 4 or 5 model hyetographs of storm rainfall have been established. Explanations: A – frequency of occurrence of storm rainfalls (1/year), B – mean duration of storm rainfalls (h/year), C – mean depth (total) of storm rainfalls (mm/year). The principle of chart construction is similar to Fig. S9, with the exception of taking into account outliers. Box notches represent the 95% confidence interval for the median.

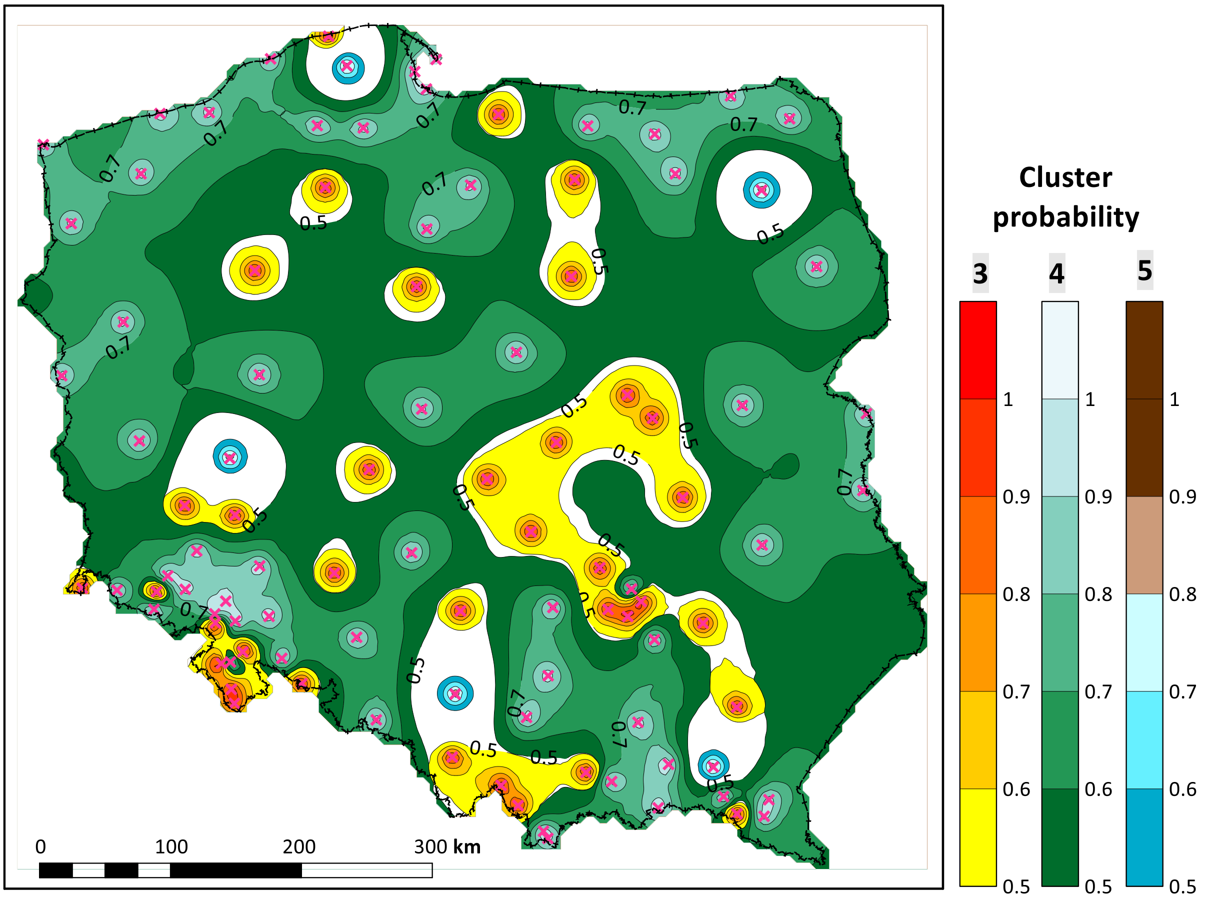

Fig. S17 Probability of membership of land in clusters with three, four, and five model hyetographs determined by means of Indicator Kriging. Colours denote probability of membership in a given cluster, if it is higher than 0.5. White (empty) spaces denote areas with the highest uncertainty, i.e. the probability of membership in any cluster is not higher than 0.5.

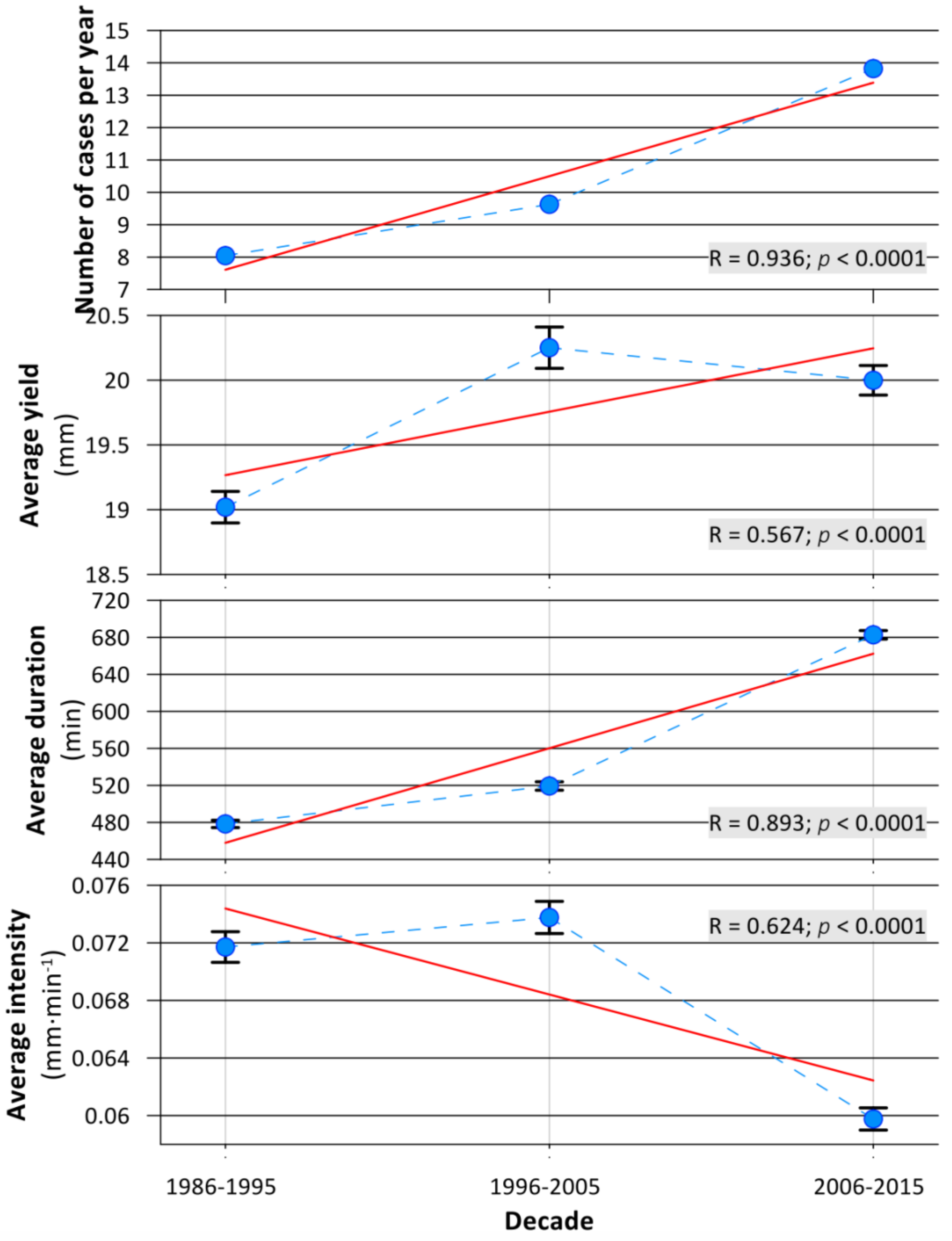

Fig. S18 Average number of cases, depth, duration and intensity of heavy rainfall in three decades from 1986 to 2015 and their standard errors. Earlier years were included in the first decade, and later years were included in the third decade. The best-fitting trend line is marked on the charts and its coefficient of determination and significance level are given.

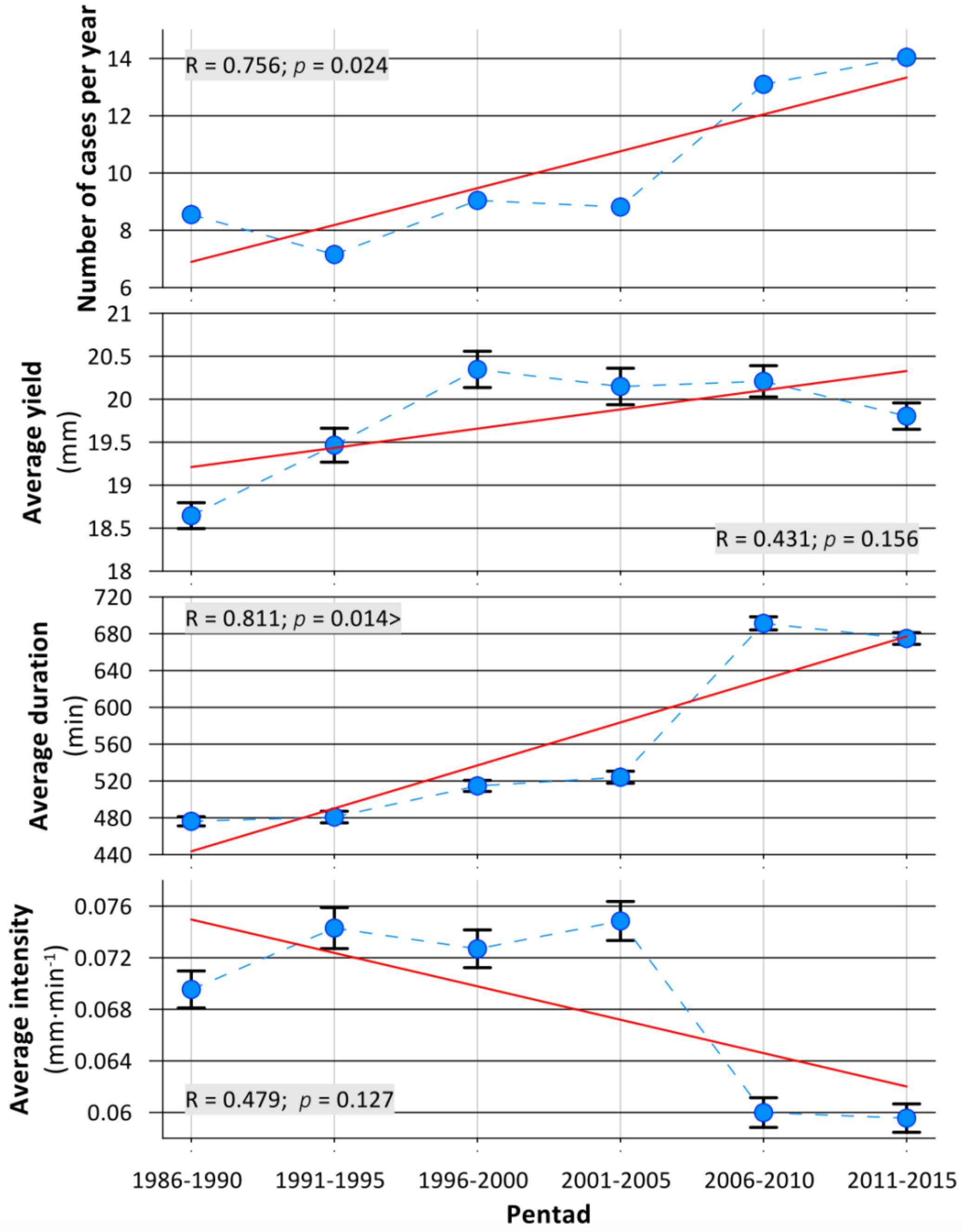

Fig. S19 Average number of cases, depth, duration and intensity of heavy rainfall in six pentads of the multiannual period 1986-2015 and their standard errors. Earlier years were added to the first pentad, and later years to the last one. The best-fitting trend line is marked on the charts and its coefficient of determination and significance level are given.

Table S1 The PANDa project stations with their location information, and statistics of the designated groups of storm rainfalls and identified clusters of model hyetographs.

No.	Name of station	Latitude	Longitude	Altitude, m a.s.l.	Number of storm rainfalls (except the removed ones)	Number of removed storm rainfalls	Number of clusters k	Minimum bootmean value
1	Baligród Mchawa	49°21′14″	22°17′01″	430	472	0	4	0.64
2	Bardo	50°30′33″	16°44′39″	320	288	0	3	0.83
3	Białystok	53°06′26″	23°09′44″	148	283	1	4	0.74
4	Biebrza	53°39′02″	22°34′42″	115	207	0	5	0.62
5	Biecz-Grudna	49°44′07″	21°17′44″	285	309	0	4	0.64
6	Bielsko-Biała	49°48′29″	19°00′04″	390	588	0	3	0.84
7	Bodzentyn	50°56′49″	20°57′05″	270	270	0	4	0.70
8	Bogatynia	50°53′59″	14°58′50″	295	308	0	3	0.87
9	Bolków	50°55′30″	16°05′55″	310	292	0	4	0.75
10	Chojnice	53°42′55″	17°31′57″	164	287	0	3	0.84
11	Chwałkowice	51°26′54″	16°36′47″	180	224	0	3	0.84
12	Cisów	50°45′31″	20°54′10″	326	252	0	3	0.86
13	Częstochowa	50°48′43″	19°05′30″	294	349	0	3	0.94
14	Długopole	50°14′59″	16°38′00″	365	330	0	3	0.94
15	Dobrogoszcz	50°45′31″	17°01′02″	175	259	0	4	0.72
16	Dzierżoniów	50°43′11″	16°39′10″	260	285	0	4	0.68
17	Elbląg	54°13′24″	19°32′37″	189	312	0	3	0.82
18	Gdańsk - Port Północny	54°23′59″	18°41′52″	2	252	0	4	0.86
19	Gdynia	54°31′08″	18°33′34″	2	217	0	4	0.70
20	Głuchołazy	50°18′08″	17°23′14″	350	325	0	3	0.83
21	Gołdap	54°18′31″	22°16′14″	160	271	0	4	0.71
22	Gorzów	52°44′28″	15°16′38″	71	226	0	4	0.78
23	Grudziądz	53°26′15″	18°42′34″	25	220	0	4	0.79
24	Hala Gąsienicowa	49°14′38″	20°00′21″	1523	726	0	4	0.69
25	Hel	54°36′13″	18°48′43″	1	271	1	4	0.61
26	Jabłonka	49°28′20″	19°41′45″	614	389	1	3	0.90
27	Jelenia Góra	50°54′01″	15°47′20″	342	309	2	3	0.92
28	Kalisz	51°46′52″	18°04′51″	137	239	0	3	0.85
29	Karpacz	50°46′44″	15°46′10″	575	398	3	4	0.74

30	Katowice	50°14′26″	19°01′58″	278	377	0	5	0.65
31	Kętrzyn	54°04′02″	21°22′00″	106	292	0	4	0.66
32	Kielce	50°48′38″	20°41′32″	260	309	0	3	0.62
33	Kłodzko	50°26′13″	16°36′51″	356	315	0	4	0.87
34	Koło	52°11′59″	18°39′37″	115	238	0	4	0.66
35	Kołobrzeg	54°10′57″	15°34′47″	3	256	0	4	0.67
36	Koszalin	54°12′16″	16°09′19″	33	381	0	4	0.65
37	Kościerzyna	54°07′43″	17°57′43″	190	286	0	4	0.71
38	Kozienice	51°33′53″	21°32′37″	123	298	0	3	0.84
39	Kraków - Bielany	50°04′40″	19°47′42″	237	320	0	4	0.66
40	Krosno	49°42′24″	21°46′09″	329	396	0	5	0.61
41	Legnica	51°11′33″	16°12′28″	122	294	0	4	0.68
42	Lesko	49°27′59″	22°20′30″	420	480	0	4	0.87
43	Leszno	51°50′08″	16°32′05″	91	270	0	5	0.64
44	Lębork	54°33′11″	17°45′25″	39	365	0	5	0.62
45	Lidzbark Warmiński	54°08′09″	20°35′10″	90	274	4	4	0.73
46	Limanowa	49°41′37″	20°25′06″	515	437	0	3	0.68
47	Lubachów	50°45′50″	16°25′43″	310	349	4	4	0.60
48	Lublin	51°13′00″	22°23′35″	238	286	3	4	0.66
49	Łeba	54°45′13″	17°32′05″	2	328	0	3	0.80
50	Łódź	51°43′06″	19°23′14″	175	243	0	3	0.78
51	Miechów	50°21′46″	20°01′58″	299	300	0	4	0.63
52	Międzylesie	50°09′12″	16°40′15″	450	395	0	3	0.88
53	Mikołajki	53°47′21″	21°35′23″	127	293	0	4	0.68
54	Mława	53°06′15″	20°21′40″	147	291	0	3	0.85
55	Namysłów	51°03′59″	17°43′00″	150	200	0	3	0.65
56	Niezabyszewo	54°08′18″	17°25′24″	160	278	0	4	0.70
57	Nowy Sącz	49°37′38″	20°41′19″	292	406	0	4	0.74
58	Olsztyn	53°46′07″	20°25′17″	133	279	2	3	0.92
59	Opole	50°37′37″	17°58′08″	163	286	0	4	0.85
60	Otmuchów	50°28′20″	17°09′59″	210	307	0	4	0.71
61	Piła	53°07′50″	16°44′50″	72	251	0	3	0.79
62	Płock	52°35′18″	19°43′33″	106	222	0	4	0.69

63	Polanica Zdrój	50°25′31″	16°31′06″	390	296	0	3	0.64
64	Polkowice	51°30′02″	16°03′22″	160	207	0	3	0.87
65	Poznań	52°25′00″	16°50′05″	88	201	0	4	0.59
66	Prabuty	53°44′22″	19°12′54″	100	269	0	4	0.63
67	Pszenno	50°51′14″	16°32′35″	225	270	0	4	0.61
68	Racibórz	50°03′40″	18°11′27″	206	281	0	4	0.64
69	Resko	53°45′49″	15°23′36″	52	326	6	4	0.77
70	Rzeszów - Jasionka	50°06′39″	22°02′32″	206	339	0	3	0.84
71	Sandomierz	50°41′48″	21°42′57″	217	304	0	3	0.88
72	Siedlce	52°10′52″	22°14′41″	152	273	0	4	0.73
73	Skierniewice	51°57′55″	20°09′37″	128	257	0	3	0.79
74	Słubice	52°20′55″	14°37′11″	53	277	0	4	0.60
75	Staszów	50°35′42″	21°11′06″	219	254	0	4	0.65
76	Sulejów	51°21′12″	19°51′59″	188	303	0	3	0.91
77	Suwałki	54°07′51″	22°56′56″	184	268	0	4	0.75
78	Szczecin	53°23′43″	14°37′22″	1	248	0	4	0.70
79	Świder	52°06′57″	21°14′15″	94	275	0	3	0.80
80	Świeradów Zdrój	50°53′54″	15°21′31″	500	536	0	4	0.87
81	Świerzawa	51°00′46″	15°54′10″	300	295	0	4	0.60
82	Święty Krzyż	50°51′34″	21°03′05″	575	316	0	3	0.85
83	Świnoujście	53°55′24″	14°14′32″	5	236	0	4	0.61
84	Tarnów	50°01′48″	20°59′02″	209	375	0	4	0.81
85	Terespol	52°04′43″	23°37′19″	133	263	0	4	0.81
86	Toruń	53°02′31″	18°35′44″	69	277	0	3	0.93
87	Ustka	54°35′18″	16°51′15″	3	340	2	4	0.73
88	Walim	50°42′10″	16°26′21″	490	372	0	3	0.88
89	Warszawa Bielany	52°16′53″	20°57′48″	98	214	0	3	0.86
90	Wieluń	51°12′40″	18°33′28″	200	280	0	4	0.70
91	Wisłoczek	49°30′03″	21°51′56″	520	460	0	4	0.75
92	Wisłok Wielki	49°22′43″	21°59′57″	540	473	0	3	0.87
93	Włochów	51°05′56″	20°36′44″	345	299	0	3	0.86
94	Włodawa	51°33′12″	23°31′46″	177	254	0	4	0.63
95	Wrocław	51°06′12″	16°54′00″	120	296	0	4	0.68

96	Wymysłów	50°49′53″	20°05′38″	250	299	0	4	0.73
97	Wysowa	49°26′17″	21°10′21″	519	473	0	4	0.7
98	Zakopane	49°17′38″	19°57′37″	855	642	0	4	0.82
99	Zawoja	49°36′43″	19°31′07″	697	580	0	3	0.84
100	Zielona Góra	51°55′48″	15°31′29″	192	258	0	4	0.75

Table S2 The PANDa project stations' statistics of the designated set of storm rainfalls of the summer half-year.

No.	Name of station	Number of observation years	Number of storm rainfalls of the summer half - year	Frequency of occurrence of storm rainfalls of the summer half - year per 1 year	Means duration of storm rainfalls of the summer half - year in a year, h	Mean depth of storm rainfalls of the summer half - year in a year, mm
1	Baligród Mchawa	30	432	14.4	126.0	308.2
2	Bardo	30	274	9.1	74.8	194.0
3	Białystok	30	250	8.3	74.1	161.6
4	Biebrza	31	196	6.3	44.1	109.6
5	Biecz-Grudna	30	288	9.6	82.4	197.8
6	Bielsko-Biała	30	532	17.7	197.9	401.2
7	Bodzentyn	30	250	8.3	60.9	157.8
8	Bogatynia	30	284	9.5	80.9	208.7
9	Bolków	30	269	9.0	79.4	193.4
10	Chojnice	30	237	7.9	71.4	146.7
11	Chwałkowice	32	219	6.8	56.0	135.0
12	Cisów	30	238	7.9	60.3	149.8
13	Częstochowa	30	307	10.2	102,.5	203.5
14	Długopole	30	315	10.5	84.4	210.7
15	Dobrogoszcz	30	239	8.0	61.4	169.1
16	Dzierżoniów	30	265	8.8	74.7	193.7
17	Elbląg	30	266	8.9	85.3	160.1
18	Gdańsk - Port Północny	30	232	7.7	59.3	145.2
19	Gdynia	30	205	6.8	53.1	119.6
20	Głuchołazy	30	288	9.6	92.4	217.0
21	Gołdap	30	249	8.3	61.2	147.6
22	Gorzów	30	189	6.3	53.3	113.0

23	Grudziądz	30	211	7.0	49.6	124.3
24	Hala Gąsienicowa	30	675	22.5	260.2	667.2
25	Hel	30	226	7.5	71.8	146.8
26	Jabłonka	30	356	11.9	108.1	233.5
27	Jelenia Góra	30	291	9.7	91.0	198.5
28	Kalisz	30	213	7.1	63.5	126.2
29	Karpacz	30	369	12.3	115.1	274.5
30	Katowice	30	329	11.0	117.5	220.7
31	Kętrzyn	30	264	8.8	78.0	155.1
32	Kielce	30	273	9.1	84.4	183.4
33	Kłodzko	30	284	9.5	98.1	198.7
34	Koło	30	209	7.0	59.5	126.3
35	Kołobrzeg	30	245	8.2	69.5	160.7
36	Koszalin	30	315	10.5	104.6	203.4
37	Kościerzyna	30	262	8.7	70.5	159.8
38	Kozienice	30	256	8.5	84.4	156.8
39	Kraków - Bielany	30	290	9.7	80.9	190.7
40	Krosno	30	352	11.7	131.9	248.4
41	Legnica	30	274	9.1	89.4	192.1
42	Lesko	30	423	14.1	137.2	291.5
43	Leszno	30	243	8.1	71.1	151.8
44	Lębork	30	298	9.9	107.6	188.7
45	Lidzbark Warmiński	30	241	8.0	59.1	145.9
46	Limanowa	30	409	13.6	131.3	297.2
47	Lubachów	30	323	10.8	89.2	238.9
48	Lublin	30	260	8.7	72.4	158.5
49	Łeba	30	284	9.5	101.9	173.2
50	Łódź	30	203	6.8	64.2	125.3
51	Miechów	30	283	9.4	77.4	182.1
52	Międzylesie	30	342	11.4	94.9	224.8
53	Mikołajki	30	266	8.9	69.0	166.6
54	Mława	30	252	8.4	73.1	153.1
55	Namysłów	30	192	6.4	52.7	120.1

56	Niezabyszewo	30	245	8.2	66.5	152.2
57	Nowy Sącz	30	373	12.4	119.6	258.0
58	Olsztyn	30	227	7.6	66.2	142.5
59	Opole	30	258	8.6	82.6	167.9
60	Otmuchów	30	276	9.2	72.8	189.0
61	Piła	30	223	7.4	62.2	138.2
62	Płock	30	195	6.5	57.1	121.3
63	Polanica Zdrój	30	266	8.9	66.9	178.5
64	Polkowice	30	189	6.3	51.9	127.3
65	Poznań	32	181	5.7	44.9	105.9
66	Prabuty	30	257	8.6	61.2	154.6
67	Pszenno	30	248	8.3	66.8	177.3
68	Racibórz	30	264	8.8	83.6	172.1
69	Resko	30	274	9.1	86.2	168.0
70	Rzeszów - Jasionka	30	309	10.3	91.7	198.1
71	Sandomierz	30	280	9.3	81.6	172.1
72	Siedlce	30	232	7.7	67.5	146.0
73	Skierniewice	30	225	7.5	50.7	136.3
74	Słubice	30	233	7.8	70.5	152.2
75	Staszów	30	239	8.0	59.5	145.3
76	Sulejów	30	260	8.7	82.2	173.6
77	Suwałki	30	235	7.8	65.6	145.6
78	Szczecin	30	211	7.0	61.7	130.5
79	Świder	30	265	8.8	59.1	160.4
80	Świeradów Zdrój	30	451	15.0	148.3	327.0
81	Świerzawa	30	275	9.2	78.7	194.9
82	Święty Krzyż	30	278	9.3	74.4	179.4
83	Świnoujście	30	196	6.5	60.1	118.7
84	Tarnów	30	339	11.3	116.3	261.9
85	Terespol	30	235	7.8	73.8	154.0
86	Toruń	30	245	8.2	71.8	160.9
87	Ustka	30	297	9.9	102.7	187.1
88	Walim	30	350	11.7	104.1	266.8

89	Warszawa Bielany	30	204	6.8	49.9	125.9
90	Wieluń	30	247	8.2	72.5	155.9
91	Wisłoczek	30	403	13.4	120.1	299.8
92	Wisłok Wielki	30	436	14.5	125.4	315.7
93	Włochów	30	264	8.8	66.2	169.7
94	Włodawa	30	233	7.8	73.4	148.2
95	Wrocław	30	271	9.0	87.7	177.4
96	Wymysłów	30	282	9.4	68.2	186.0
97	Wysowa	30	456	15.2	123.8	315.3
98	Zakopane	30	568	18.9	238.5	449.4
99	Zawoja	30	501	16.7	167.2	359.8
100	Zielona Góra	30	224	7.5	73.1	146.3
